# In vivo antiangiogenic effect of nimbolide, trans-chalcone and piperine for use against glioblastoma

**DOI:** 10.1186/s12885-023-11625-4

**Published:** 2023-11-30

**Authors:** Anna Senrung, Tanya Tripathi, Joni Yadav, Divya Janjua, Apoorva Chaudhary, Arun Chhokar, Nikita Aggarwal, Udit Joshi, Nidhi Goswami, Alok Chandra Bharti

**Affiliations:** 1 Department of Zoology, Molecular Oncology Laboratory, University of Delhi (North Campus), Delhi, 110007 India; 2 Neuropharmacology and Drug Delivery Laboratory, Daulat Ram College, University of Delhi, Delhi, India; 3 Deshbandhu College, University of Delhi, Delhi, India

**Keywords:** Glioblastoma multiforme, 3D-spheroids, Chick CAM model, Angiogenesis, U87 xenograft

## Abstract

**Background:**

Angiogenesis is an important hallmark of Glioblastoma (GBM) marked by elevated vascular endothelial growth factor-A (VEGF-A) and its receptor 2 (VEGFR-2). As previously reported nimbolide (NBL), trans-chalcone (TC) and piperine (PPR) possess promising antiangiogenic activity in several cancers however, their comparative efficacy and mechanism of antiangiogenic activity in GBM against VEGFR-2 has not been elucidated.

**Methods:**

2D and 3D spheroids cultures of U87 (Uppsala 87 Malignant Glioma) were used for evaluation of non-cytotxoic dose for anti-angiogenic activity. The antiangiogenic effect was investigated by the GBM U87 cell line bearing chick CAM model. Excised U87 xenografts were histologically examined for blood vascular density by histochemistry. Reverse transcriptase polymerase chain reaction (RT-PCR) was used to detect the presence of avian and human VEGF-A and VEGFR-2 mRNA transcripts.

**Results:**

Using 2D and 3D spheroid models, the non-cytotoxic dose of NBL, TC and PPR was ≤ 11 µM. We found NBL, TC and PPR inhibit U87-induced neoangiogenesis in a dose-dependent manner in the CAM stand-alone model as well as in CAM U87 xenograft model. The results also indicate that these natural compounds inhibit the expression of notable angiogenic factors, VEGF-A and VEGFR-2. A positive correlation was found between blood vascular density and VEGF-A as well as VEGFR-2 transcripts.

**Conclusion:**

Taken together, NBL, TC and PPR can suppress U87-induced neoangiogenesis via a reduction in VEGF-A and its receptor VEGFR-2 transcript expression at noncytotoxic concentrations. These phytochemicals showed their utility as adjuvants to GBM therapy, with Piperine demonstrating superior effectiveness among them all.

**Supplementary Information:**

The online version contains supplementary material available at 10.1186/s12885-023-11625-4.

## Introduction

Glioblastoma multiforme (GBM) is the most common aggressive malignant form of brain cancer [1–3]. It accounts for 30% of all central nervous system (CNS) tumors, 45% of malignant and 80% of the primary malignant CNS tumors [4]. Though brain and CNS cancers form only about 1.6–2% of annual cancer incidence [5], they are severely notorious, life-threatening and form a significant source of cancer-related mortality and morbidity worldwide [6]. Despite intensive treatment, the survival period ranges only about 12–15 months [7]. GBM with no complete cure to date, is considered one of the deadliest human cancers [8] pose with several challenges. Amongst the several bottlenecks in the treatment of GBM, the presence of the blood–brain barrier (BBB) and neoangiogenesis are the major challenges in the treatment of GBM limiting the effective delivery of drugs for efficacy and complete cure.

GBM as a rapidly dividing solid tumor is coupled with marked angiogenesis that governs GBM wholeness [9–11]. This elevated angiogenic process in GBM and several other diseases is the outcome of increased expression of pro-angiogenic factors, viz., vascular endothelial growth factor (VEGF), basic fibroblast growth factor (bFGF), platelet-derived growth factor (PDGF), tumor necrosis factor (TNF), interleukin 8 (IL-8), transforming growth factor alpha (TGFα), and hepatocyte growth factor (HGF), over that of antiangiogenic factors, viz., angiostatin, endostatin, thrombospondin. Amongst the several pro-angiogenic signalling/regulatory pathways, the VEGF/VEGFR-2 axis is the absolute regulator of the angiogenesis process. The GBM tumor cells in principle release VEGF that directs resident endothelial cells (EC) via its receptor 2 (VEGFR 2) to form new blood vessels [12–14]. Inhibition of VEGFR-2 to block VEGF-A-regulated angiogenesis reduced tumor growth in GBM [15, 16]. Targeting of angiogenesis via inhibition of VEGFR-2 [17] coupled with the killing of GBM cells is an effective way of controlling GBM progression.

There are well-standardized antiangiogenic agents for clinical application, viz., vandetanib, sunitinib, sorafenib, everolimus, lenvatinib, pazopanib, regorafenib, cabozantinib, axitinib, bevacizumab, ramucirumab, ziv-aflibercept, and others [18]. However, their clinical usage with GBM has met with several undesirable health consequences [19–21]. Therefore, the requisite for antiangiogenic agents for GBM is still in contention. Amid reportedly potential antiangiogenic compounds, the agents of plant source with well-characterised structures and properties noted in chemical databases such as PubChem are considered promising leads that can be carried from bench to bedside translation. There are several phytochemicals reported to be antiangiogenic with no noted dreadful health ramifications. However, their ability to cross BBB in conjunction with antiangiogenic activity has not been well defined. In our unpublished in silico study, the phytochemicals, nimbolide (NBL), trans-chalcone (TC), and piperine (PPR), were predicted to have good binding affinity with VEGFR-2 tyrosine kinase domain (VEGFR-2 TKD) at the ATP binding site compared to ATP, and capable of crossing BBB using in silico tools (Supplementary Figures SF1-SF4). These phytochemicals, viz., NBL, TC, and PPR have been reported with several therapeutic usages including renoprotective, anti-cancer, anti-inflammatory, hepatoprotective, anti-diabetic, and antioxidant, but the study on their antiangiogenic property is restricted.

In the present study, we investigated the antiangiogenic properties of NBL, TC, and PPR for their application in GBM using an in vivo CAM model. CAM is one of the finest angiogenic models considering their innate highly angiogenic properties and the similarity in the network of angiogenic growth factors and receptors between developmental stages and cancer [22, 23]. Interestingly, the antiangiogenic effect of these phytochemicals has never been evaluated for GBM nor in the well-established chick CAM angiogenesis model. For the first time, using the CAM model, we have shown the antiangiogenic properties of NBL, TC, and PPR, for therapeutic application in the GBM tumour microenvironment.

## Materials and methodology

### Biological specimens and animal ethics approval

The human glioblastoma U87(MG) cell line was purchased from the National Centre for Cell Science (NCCS), Pune, India. Pathogen-free fertilized white Leghorn (*Gallus gallus*) eggs were procured from Venky’s (India) Limited, Haryana Division, India. Research on Chick CAM was conducted following Basel Declaration consensus principles and implementation of the 3Rs for the welfare of the participating embryos. The study was approved by the Institutional Animal Ethics Committee (IAEC approval ID DU/KR/IAEC/2017/06) of Daulat Ram College, University of Delhi, Delhi, India. The chick embryos were euthanized by decapitation following the American Veterinary Medical Association (AVMA) guidelines for Euthanasia of Animals.

### U87 cell culture and preparation of phytochemical stock solution

Human glioblastoma U87MG (an epithelial-like glioblastoma cell line established from patient having astrocytoma-glioblastoma) was cultured in Dulbecco’s Modified Eagle’s Medium (DMEM) added with 1% Non-Essential Amino Acid, 1 mM sodium pyruvate, 10% (v/v) fetal bovine serum, 2% Pen strep Glutamine, inside humidified CO_2_ incubator (Galaxy 170S) at standard conditions of 37 °C and 5% CO_2_. The solubility of the test phytochemicals (NBL, TC, PPR) and the reference drug AXI in aqueous solution is very poor. Therefore, these compounds were solubilized in DMSO to make a stock solution of 200 mM. The working stock of 100 µM was made in culture media. The toxicity of the solvent was pretested on cells and tissues used in various assays that were implemented in the present study**.** The final DMSO concentration was not allowed to exceed beyond the non-toxic range.

### Cell viability determination by MTT assay

U87 at a cell density of 5 × 10^3^ cells/well were seeded in triplicates in 96-well flat-bottomed culture plates. After overnight incubation, each well was replaced with fresh media containing different concentrations of test compounds and only culture media as control. After 72 h, the cell cytotoxicity dose was determined by MTT assay. For this, the cells were washed with 1 × PBS, and 100 µl of MTT reagent (50 µl of culture media and 50 µl of MTT reagent; MTT Stock = 5 mg/mL) was added to each well. The plates were incubated for 2–3 h in the CO_2_ incubator followed by the addition of 100 µl of the solubilizing solution (DMSO) in each well. The plate was then incubated at room temperature in the dark for 15 min or more till the dissolution of formazan crystal (the plate was shaken in between to help dissolve the crystals faster). The absorbance was recorded at 570 nm on the Microplate Reader.

### U87 3D spheroid generation

U87 3D spheroids were generated by 2 techniques: Hanging Drop (HD) and Liquid Overlay Technique (LOT) following the protocol described earlier [24]. For HD, 20 µl (250 cells/µl) of cell suspension was placed sufficiently apart onto the lid of the cell culture dish 100 × 20 mm on the inner surface. The lid with drops of cells was inverted and placed over the bottom chamber of the dish, containing 1 × PBS. Cells were visualized on the 5^th^ day under a microscope (10 ×). Similarly, for LOT, 5000 cells/20 µl of culture media were placed into each well of the 96-well ultra-low attachment (ULA) plate. The cultures were incubated inside a humidified CO_2_ incubator at standard conditions of 5% CO_2_ and 37 °C. The spheroids were examined on the 5^th^ day under the microscope (10 ×).

### Cell viability determination for U87MG by 3D spheroid generation as a measure of spheroid volume estimation

For this, U87 cells were incubated for 3D spheroid formation by both HD and LOT methods in different working concentrations of drugs that were prepared in culture media. The HD method was adopted from the protocol described earlier [25], and LOT was performed following the protocol modified from another study [26]. The spheroid cultures were supplemented with 10 µl of fresh media on day 2 (the first day of incubation was considered as day 0) and harvested after 4 days (or day 5) and the size/volume was determined using the standard practice formula: volume (mm^3^) = 4/3π × r^3^, where, r = L/2 or (L + W)/2, L = major anteroposterior diameter, W = diameter from left to right [27]. The study was performed in triplicates for each drug concentration in three independent experiments.

### Validation of U87-3D-spheroid cell viability with PI & DAPI staining

On day 5 (96 h) post U87 3D spheroid incubation with test compounds for spheroid formation, a set of spheroids (from each drug concentration) was stained with propidium iodide (PI) while another set was stained with 4’,6-diamidino-2-phenylindole (DAPI). For this 5 µl of PI (10 mg/ml PBS) and DAPI solution (5 µg/ml PBS) were added to each spheroid culture (HD & LOT). The mixture was incubated in the dark at room temperature for about 20–25 min. Positive spheroids were observed under the microscope and images were taken.

### Chick CAM angiogenesis assay

CAM angiogenesis assay was performed as described by Ribatti et al. [28] with modifications using a polystyrene boat [29]. Briefly, gelatin sponges (GS) of about 6.6 mm × 6.6 mm × 3.5 mm (L x B x thickness), pre-soaked in different concentrations of test compounds (~ 60 µl) were implanted onto the CAM on CAM on embryonic development day 9 (EDD9), Hamburger-Hamilton stage 34 (HH stage 34). After 48 h, the images of the experimental CAM were taken and vascularization density was analysed for the quantitative determination of the antiangiogenic effect of the phytochemicals through the number of nodes, meshes and total length at the experimental site with ImageJ version 1.52a [30].

### Chick CAM U87-xenograft angiogenesis assay

For this study, following the method used by Chen et al. [31] with minor modifications in the windowing method [32], 30 µl of U87 cell suspension containing 1.5 million cells supplemented with different concentrations of test compounds were loaded onto GS (L x B, 6.6 mm × 6.6 mm; thickness, 3.5 mm) and placed on chick CAM of EDD9. Parallelly, a set of chick CAMs was normally grown/incubated with no alterations which was taken as negative control, while another set was implanted with U87 cells without treatment for positive control. On EDD12 (HH stage 38), the U87 xenografts were photographed and analysed for blood vascular density on CAM around the tumor cell implantation site using ImageJ version 1.52a for the quantitative determination of the antiangiogenic effect of the phytochemicals through the number of nodes, meshes and total length formed. The xenografts were further excised, weighted, and blood vascular density within the tumor xenograft was analysed.

### Determination of blood vascular density within the tumor xenografts

U87 xenografts from each treatment concentration fixed in 10% formalin were processed for haematoxylin and eosin (H&E) staining following the protocol modified from the earlier described study [33]. Briefly, the xenografts were dehydrated by passing through a series of graded alcohols, embedded in low-melting-point paraffin and sectioned at a thickness of 5 µm. Tissue sections were then stained with H&E and evaluated for blood vessels. Blood vessels were manually recorded for each tumor section using Nikon Eclipse Ts2R (Nikon, USA). Analysis was carried out on three randomly selected fields of high blood density at a magnification of × 20. The blood vessels were further indicated and validated by Tomato lectin Dylight 594 (TL-DL594) histochemistry and immunohistochemistry (IHC) for desmin. TL-DL594 histochemistry was performed as described by Villacampa et al. [34] with few modifications. Briefly, the sections after deparaffinization were incubated with endogenous peroxidase blocking solution (EPBS) at room temperature (RT) for 10 min and then washed with tris-buffered saline (TBS) with triton X-100 (TBST), incubated with TL-DL594 diluted in TBST (1:1000) for 2 h at RT, washed and analysed by fluorescence microscopy. For desmin IHC the tissue sections after deparaffinization and antigen retrieval were incubated for 1 h at RT with primary antibody (Mouse IgG1/K), washed with TBS buffer and then incubated with Horseradish Peroxidase for 30 min at RT, washed and stained with 3,3′-Diaminobenzidine (DAB) for 5 min. Lastly, counterstaining was performed with hematoxylin and observed with light microscopy.

### Determination of gene transcript level changes by Reverse Transcriptase-PCR (RT-PCR)

RNA from control and treated CAM tissues and CAM U87 xenografts were isolated using TRIzol RNA isolation reagent as described earlier [35, 36]. Quantification of isolated RNA was performed by Nanodrop One (Thermo Scientific, USA). Complementary DNA (cDNA) was prepared from 10 µg of RNA sample in a 20 µl reaction using a High-Capacity verso cDNA synthesis kit (Thermo Scientific, USA). For amplification of the VEGFR-2 gene, polymerase chain reaction (PCR) was performed on Biometra Tadvanced (Germany) in a 25 µl reaction system. The PCR program consisted of a holding stage of 5 min at 95ºC, 40 cycles of denaturation at 95ºC for 10 s, annealing at 60ºC for 15 s, followed by polymerization at 72ºC for 10 s and final extension at 65ºC for 1 min. The primer sequence with annealing temperature is given in Supplementary Table 1. GAPDH was used as internal control and all the quantifications were normalized to the level of GAPDH transcripts.

### Statistical analysis

Curve fitting and statistical analysis were done using Microsoft Excel (2010) and the GraphPad Prism-5 software, San Diego, CA. All the data are represented as mean ± standard deviation of the mean. The dose–response curve was measured by Student’s t-test. For comparison of the mean between two groups, a two-tailed unpaired t-test was performed and for mean across multiple groups (> 2), one-way ANOVA followed by post-hoc Tukey’s multiple comparison analysis was performed. *P* value < 0.05 was considered significant and levels of statistical significance *p < 0.05, **p < 0.01, ***p < 0.001, are represented and indicated with respective graphs.

## Results

### Determination of U87 cell viability by MTT

**Fig. 1:**
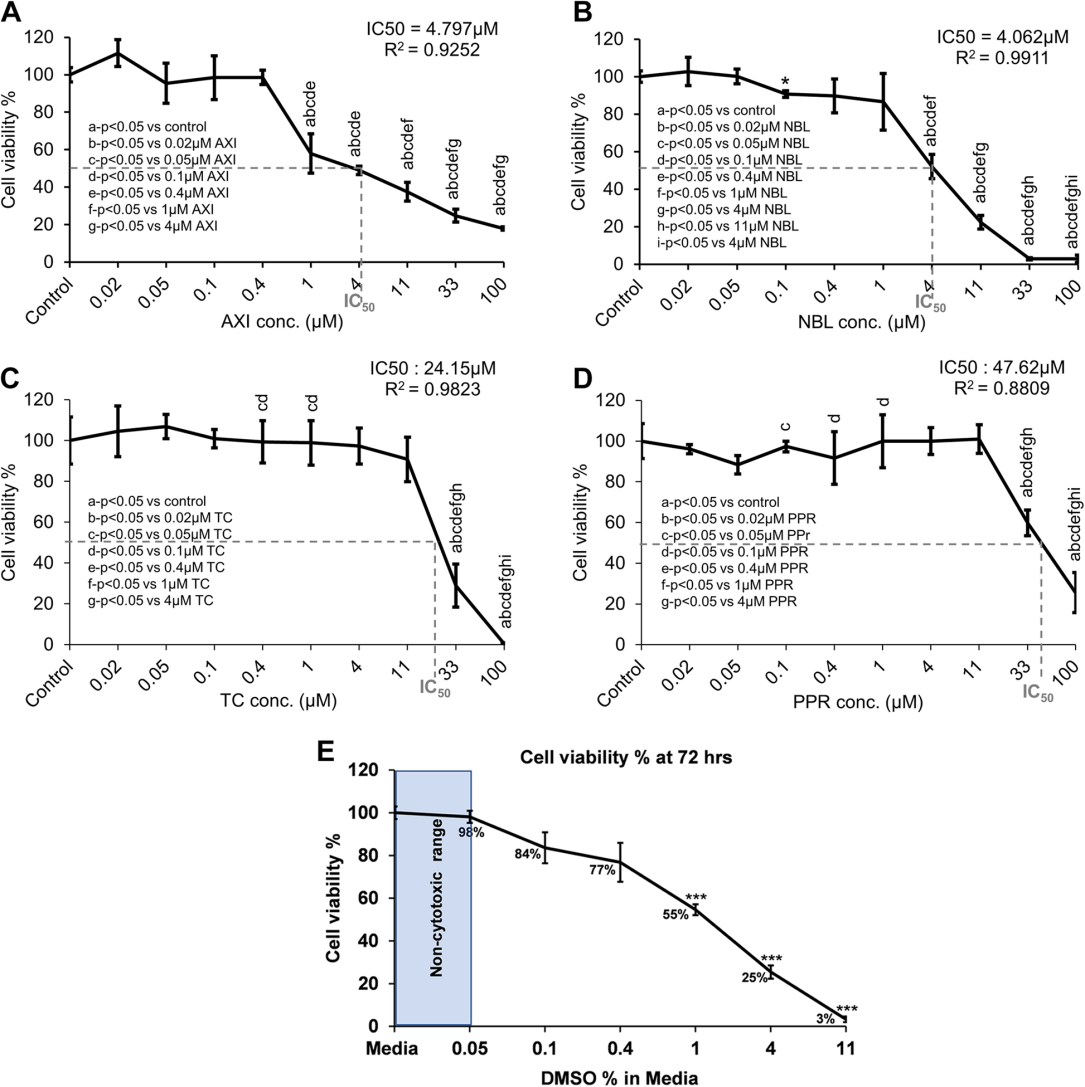
Cytotoxicity dose of AXI, NBL, TC and PPR determined for U87 cells by MTT assay. **A** AXI; **B** NBL; **C** TC; **D** PPR. **E** DMSO. DMSO-Dimethyl sulfoxide; AXI-Axitinib; NBL-Nimbolide; TC-Trans-Chalcone; PPR-Piperine, *p*-value < 0.05 was considered significant

In the first phase of the in vitro study, the optimum non-cytotoxic concentration of NBL, TC, PPR, and the reference antiangiogenic agent, axitinib (AXI) was determined by U87 cell viability assay. The direct influence of AXI, NBL, TC and PPR was measured at concentrations ranging from 0.02–100 µM using the cellular methylthiazoltetrazolium (MTT)-cleavage activity of U87. Cells showed a dose-dependent growth inhibition response against all the agents used, however, the inhibition kinetic differed for each agent (Fig. 1). Half-maximum inhibitory concentrations (IC50) recorded at 72 h were 4.797, 4.062, 24.15 and 47.62 µM for AXI, NBL, TC and PPR, respectively. As we planned to use AXI as a positive control as an angiogenic inhibitor, we aimed to find a concentration of AXI and other phytochemicals where they exert a minimal direct effect on cell viability and identified a concentration where the growth inhibitory effect is minimal. AXI treated U87 cells exhibited > 50% (Mean ± SD, 57.95 ± 10.54) cell viability at 1 µM whereas it was > 80% at ≤ 0.4 µM. NBL treated U87 cells exhibited > 50% (Mean ± SD, 52.12 ± 6.54) cell viability at 4 µM and > 80% at ≤ 1 µM. TC and PPR treated cells exhibited ≥ 80% cell viability at concentrations ≤ 11 µM. Taken together, keeping < 20% growth inhibition as cut-off, TC and PPR exhibited moderate cytotoxic effects on U87 cells at below 11 µM concentration in 2D cultures. Vehicle (DMSO) did not show any cytotoxic effect up to 0.05% **(**Fig. 1E**)**. There was a moderate decline in cell viability up to 0.4% DMSO, however, it was not statistically significant.

### U87 cell viability determination by 3D spheroid generation

**Fig. 2:**
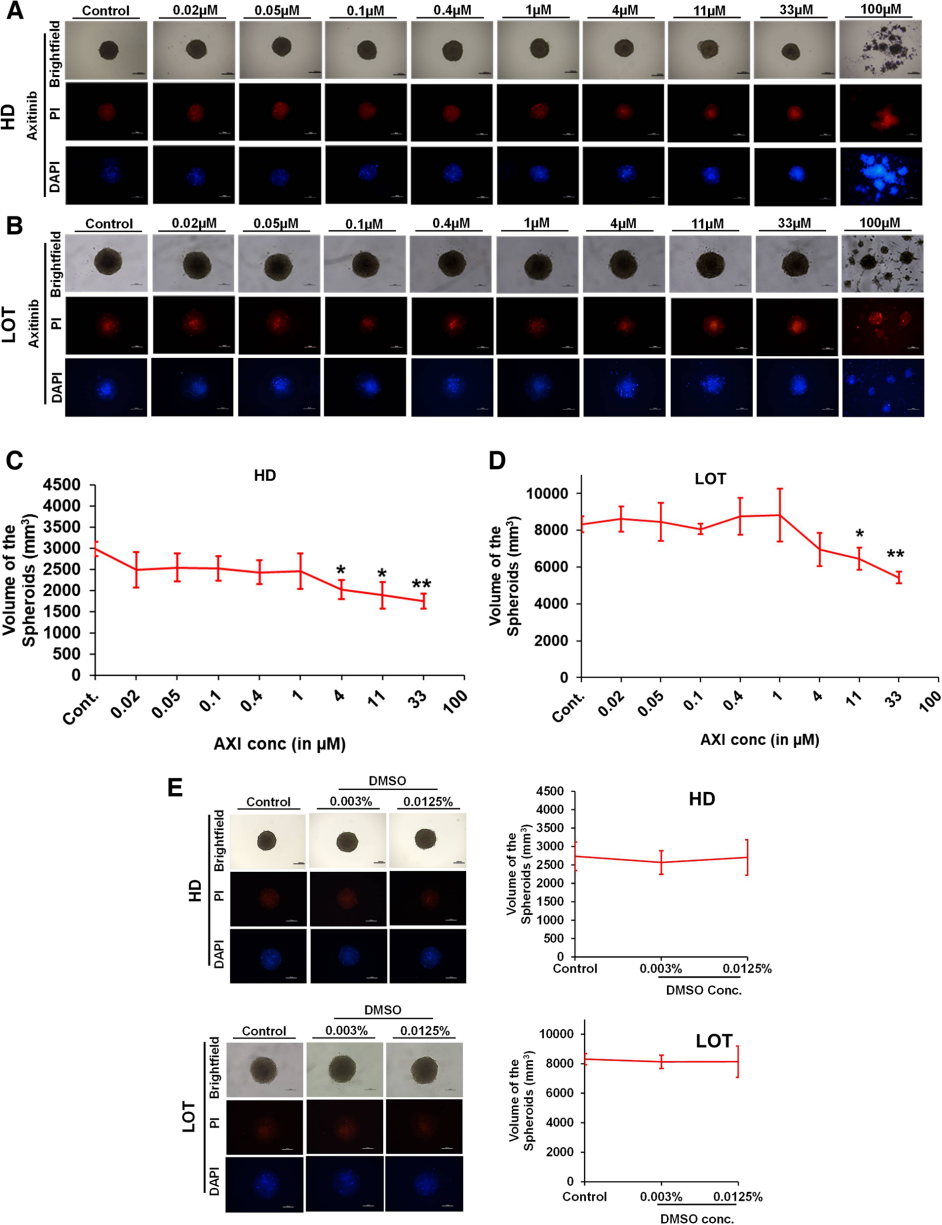
Effect of AXI on U87 cell viability determined by 3D spheroid generation. **A-B** Representative images of spheroids from different concentrations of AXI shown in brightfield (upper row), red fluorescence with PI (middle row), blue fluorescence with DAPI (low row) generated by HD and LOT; **C-D** Graphical representation of the spheroid volumes obtained from cells supplemented with variable doses of AXI where spheroid integrity has been retained. **E** Effect of vehicle control (DMSO) on U87 cell viability of 3D spheroids. *, ** = significant with *p*-value < 0.05 & < 0.001 respectively. Abbreviations: DMSO-Dimethyl sulfoxide; HD-Hanging drop Method; LOT-Liquid overlay technique; PI-Propidium iodide; DAPI-4’,6-diamidino-2-phenylindole. Scale bar-100 µm

Further we checked the effect of AXI, NBL, TC and PPR on the growth and viability of U87 cells in 3D spheroids. The spheroids were generated in the presence of different concentrations of AXI (standard antiangiogenic reference drug) and other lead compounds both by HD and LOT methods. The cells were incubated for 4 days (96 h) and terminated on day 5 as the cells around this time form definite spheroids in control/normal culture. Morphometric evaluation of cytotoxic effect including a reduction in spheroid size, disruption of spheroid integrity, segregation of spheroidal cells into smaller aggregates, loosely dispersed or scattered cells, were observed only at concentrations greater than IC50 (in 2D culture) of test compounds on U87 cells. AXI at test concentrations ≥ 4 µM showed a significant (*p*-value < 0.05) reduction in spheroid volume compared to control and exhibited small dispersed aggregates of cells at 100 µM (Fig. 2). Vehicle control DMSO at 0.003% and 0.0125% concentrations corresponding to 1 and 11 µM test phytochemical concentrations did not show any significant difference in 3D spheroid volume and morphology (Fig. 2E).

**Fig. 3:**
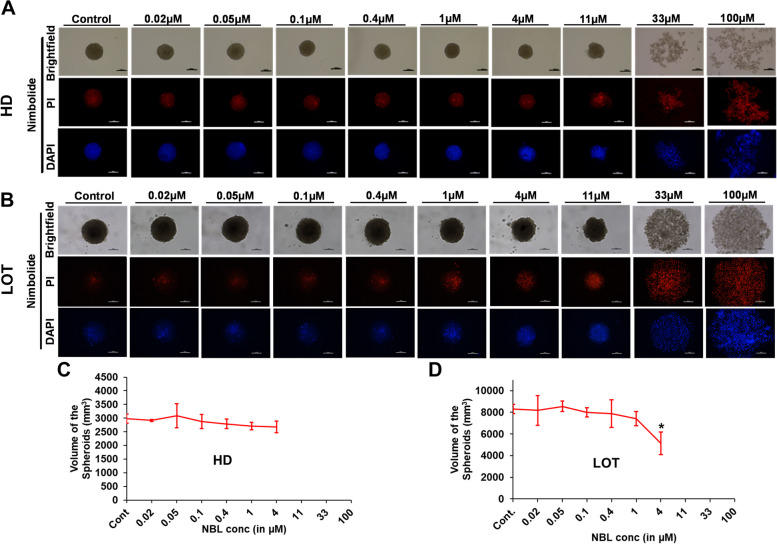
Effect of NBL on U87 cell viability determined by 3D spheroid generation. **A-B** Representative images of spheroids from different concentrations of NBL are shown in brightfield (upper row), red fluorescence with PI (middle row), blue fluorescence with DAPI (low row) generated by HD and LOT; **C-D** Graphical representation of the spheroid volumes obtained from cells supplemented with variable doses of NBL where spheroid integrity has been retained. * = significant with *p*-value < 0.05. Abbreviations: HD-Hanging drop Method; LOT-Liquid overlay technique; PI-Propidium iodide; DAPI-4’,6-diamidino-2-phenylindole. Scale bar-100 µm

**Fig. 4:**
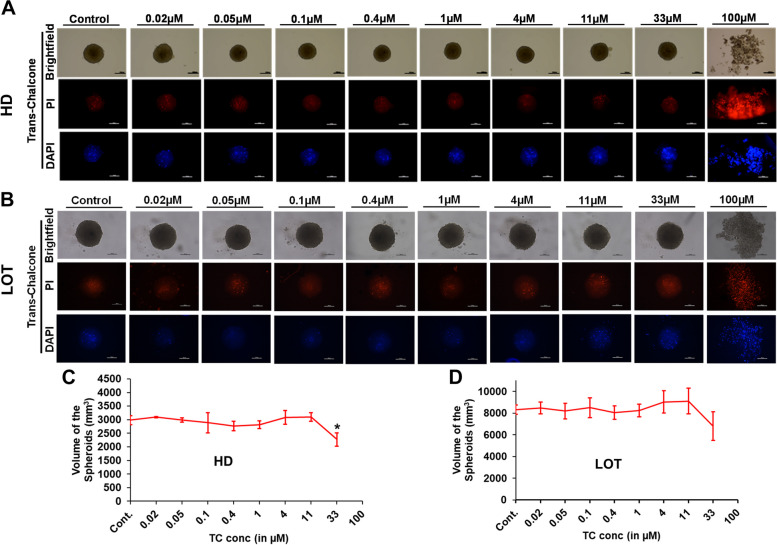
Effect of TC on U87 cell viability determined by 3D spheroid generation. **A-B** Representative images of spheroids from different concentrations of TC shown in brightfield (upper row), red fluorescence with PI (middle row), blue fluorescence with DAPI (low row) generated by HD and LOT; **C-D** Graphical representation of the spheroid volumes obtained from cells supplemented with variable doses of TC where spheroid integrity has been retained. * = significant with *p*-value < 0.05. Abbreviations: HD-Hanging drop Method; LOT-Liquid overlay technique; PI-Propidium iodide; DAPI-4’,6-diamidino-2-phenylindole Scale bar-100 µm

**Fig. 5:**
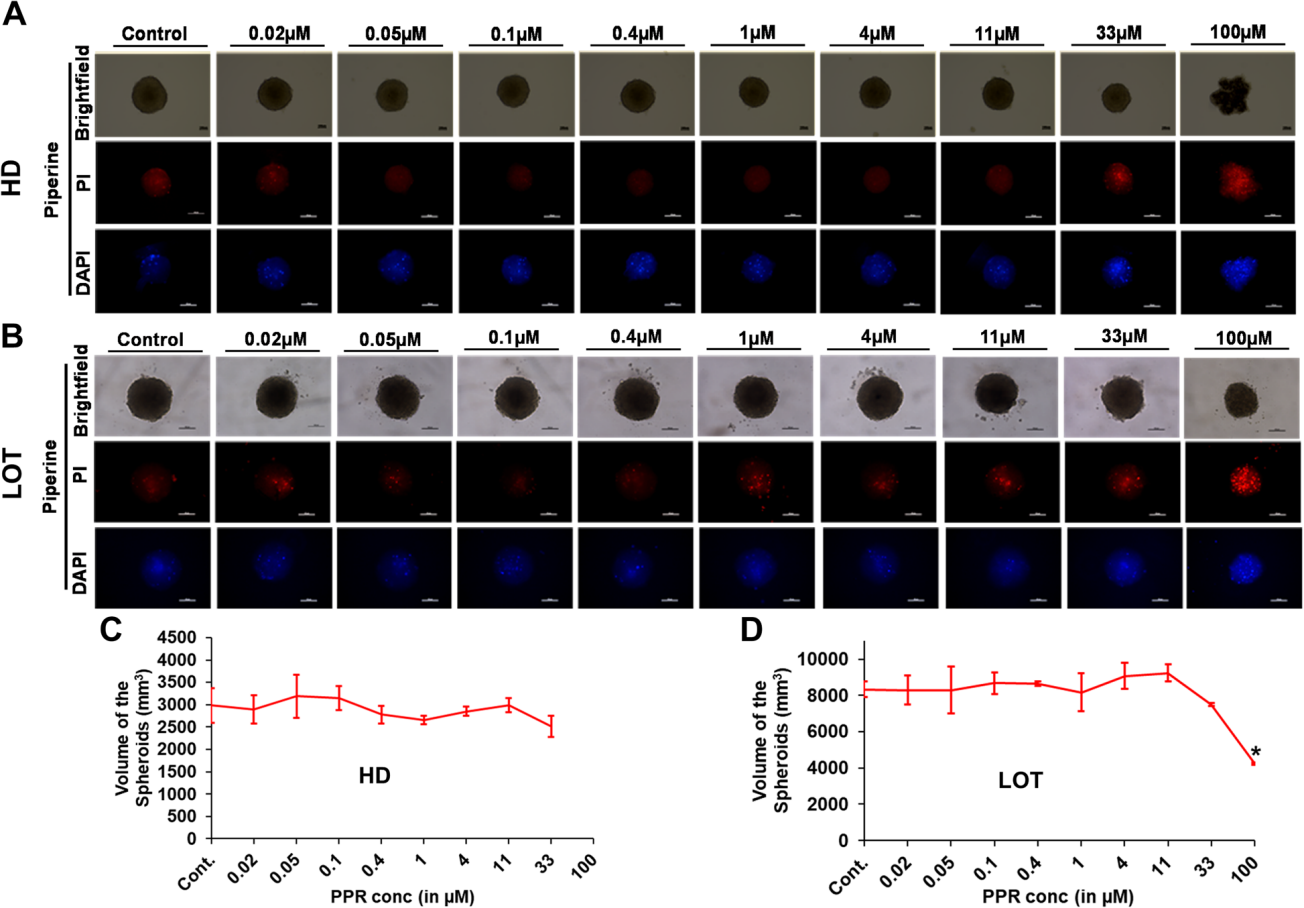
Effect of PPR on U87 cell viability determined by 3D spheroid generation. **A-B** Representative images of spheroids from different concentrations of PPR are shown in brightfield (upper row), red fluorescence with PI (middle row), blue fluorescence with DAPI (low row) generated by HD and LOT; **C-D** Graphical representation of the spheroid volumes obtained from cells supplemented with variable doses of PPR where spheroid integrity has been retained. * = significant with *p*-value < 0.05. Abbreviations: HD-Hanging drop Method; LOT-Liquid overlay technique; PI-Propidium iodide; DAPI-4’,6-diamidino-2-phenylindole. Scale bar-100 µm

NBL exhibited a reduction in spheroid volume at 4 µM only in LOT and dysregulated spheroidal integrity as well as loosened cells at concentrations > 4 µM (Fig. 3). In the case of TC and PPR cytotoxic effects on spheroid integrity/morphology appeared at 33 µM and prominent only at 100 µM (Figs. 4 and 5). Irrespective of the spheroid volume size, spheroid integrity was retained in all the treatments at concentrations ≤ 11 µM inferring a good degree of cell viability at these concentrations. Apart from NBL, all the other three compounds, AXI, TC and PPR, retained spheroid integrity up to 33 µM. Further, the cell viability in 3D spheroids was validated by dead cell staining of PI (red fluorescence) and DAPI (blue fluorescence).

### Antiangiogenic activity of NBL, TC and PPR on chick CAM

**Fig. 6:**
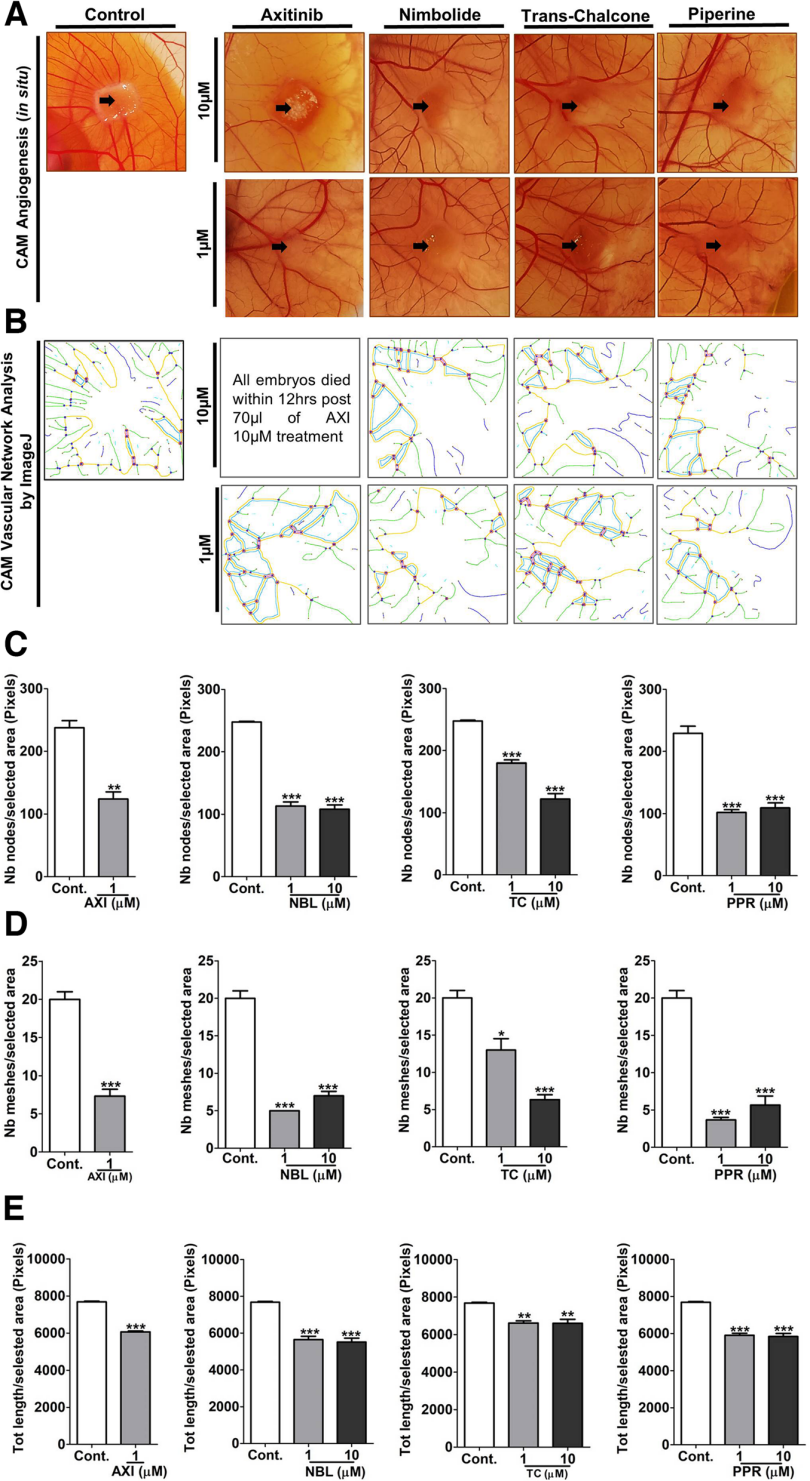

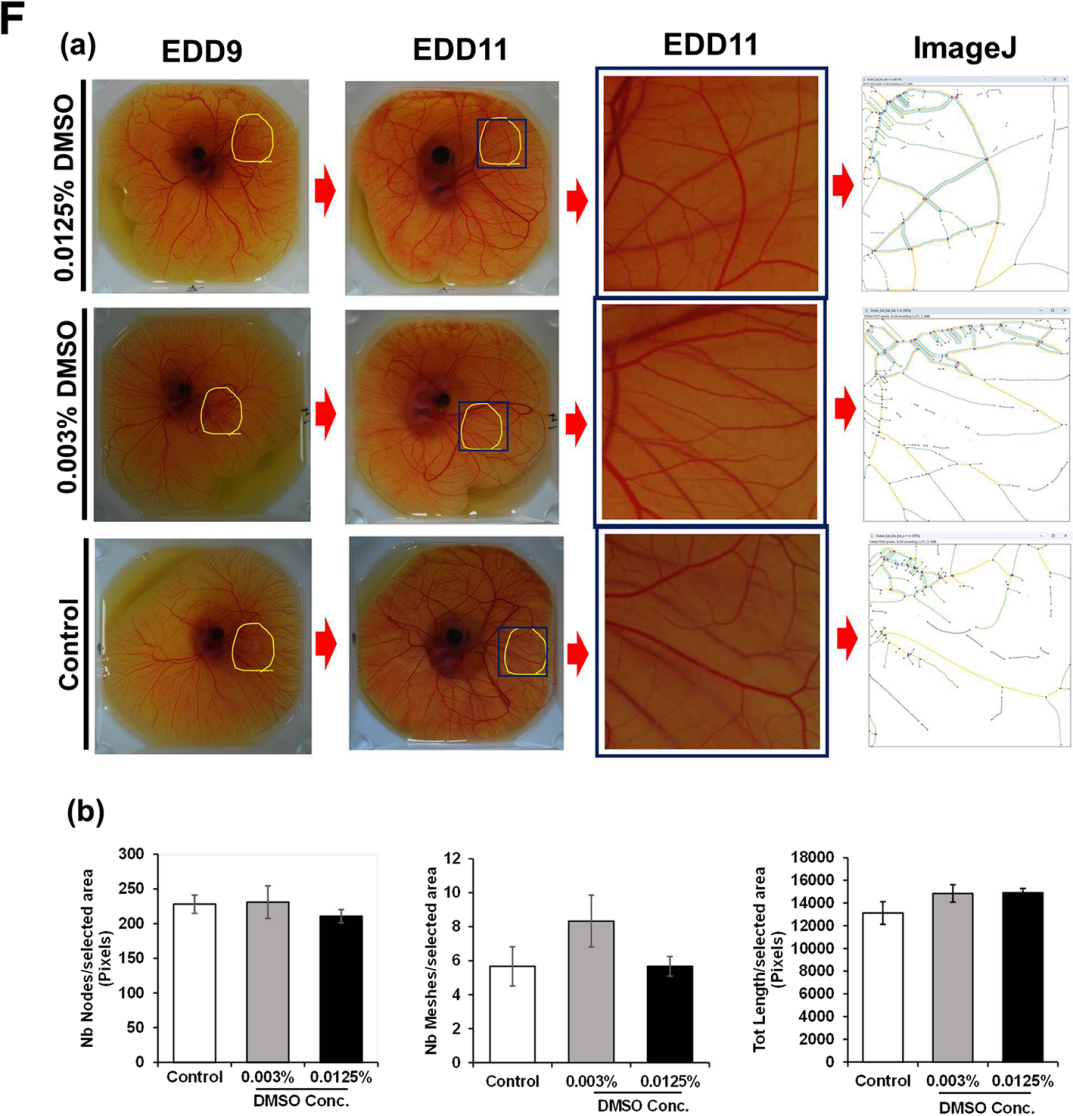
Antiangiogenic effect of AXI, NBL, TC and PPR on Chick CAM Neoangiogenesis. **A** Photographic representation of the antiangiogenic effect of AXI, NBL, TC and PPR at 10 µM (upper row) and 1 µM (lower row) and control (1 × PBS); **B-E** Quantitative analysis of antiangiogenic properties of AXI, NBL, TC and PPR at doses 10 and 1 µM in comparison to control (1 × PBS). **F** Effect of vehicle alone (DMSO) at corresponding concentration in test phytochemicals on chick CAM Neoangiogenesis. Treatment was given on EDD9 and incubated for 48 h (EDD11). Images captured through Nikon DSLR were analysed for the macro-vesicular organisation by quantifying nodes, meshes and the total length of blood vessels around the site of drug administration using ImageJ version 1.52a. Statistically, the antiangiogenic analysis for AXI, NBL, TC and PPR indicates a significant decrease in the nodes, meshes and length of blood vessels in the treated in dose-dependent manner compared to control, however, the antiangiogenic analysis for DMSO at both concentrations did not show any significant difference from control. Drastic effects would be observed if microvasculatures were visualised and captured. **, *** = significant with *p*-value < 0.001 & < 0.0001 respectively. CAM-Chorioallantoic membrane, EDD-Embryonic Development Day, DMSO-Dimethyl sulfoxide

To validate NBL, TC and PPR as potential lead antiangiogenic compounds in vivo, a chick CAM angiogenesis assay was performed. For the assay, a dose of 1 and 10 µM of each test compound were evaluated at 48 h post-treatment on EDD11. AXI was used as a positive control. Both qualitatively and quantitatively, CAM exhibited antiangiogenic characteristics in response to different doses of AXI, NBL, TC and PPR concentrations (Fig. 6A-B). All treated chick embryos survived till the termination of the assay (at 48 h, EDD11) except for those treated with 10 µM AXI. The chick embryos treated with 10 µM AXI died within 12 h of drug treatment with reduced vasculature around the drug application site. Interestingly, embryos treated with 10 µM AXI on EDD10 survive beyond 12–15 h. We hypothesize, the stability of the vascular network. Qualitatively CAM exhibited vessel disorganization and tortuosity, in response to AXI, NBL, TC and PPR at both dosages (Fig. 6C-E). Quantitatively using ImageJ, a significant decrease in the above parameters representing reduced blood vascularity, namely the nodes, meshes and total length in CAM vasculature was observed at both treated doses in comparison to the negative control (PBS). These changes were comparable to AXI. All the test compounds exhibited a significant inhibitory effect on CAM vasculature compared to negative control (1 × PBS). DMSO at 0.003% and 0.0125%, corresponding to test phytochemical concentrations 1 and 10 µM, did not show any change in the nodes, meshes, and length of blood vessels in comparison to the control (1 × PBS) (Fig. 6F).

### Effect of NBL, TC and PPR on VEGF-A and VEGFR-2 transcript in chick CAM

**Fig. 7:**
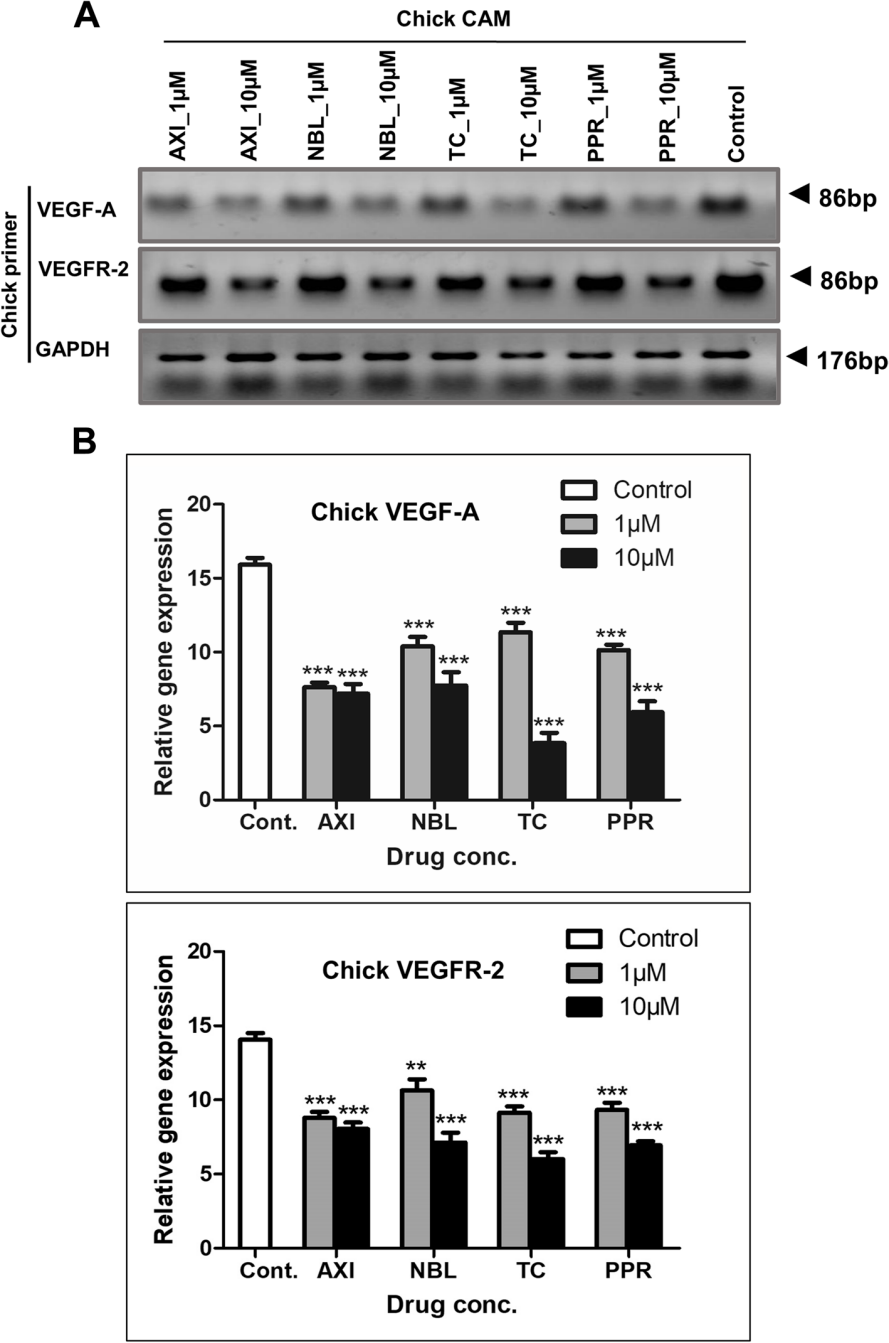
Gene transcript expression level for treated and control Chick CAM. **A** Gel images showing transcript expression of VEGF-A and VEGFR-2 in chick CAM (Full gel images included in Supplementary Information File); **B** Densitometric evaluation of VEGF-A & VEGFR-2 gene level expression in CAM relative expression with respect to GAPDH transcript expression. **, *** = significant with *p*-value < 0.001 & < 0.0001 respectively

Amongst proangiogenic factors, VEGF-A and its receptor VEGFR-2 are the most notable factors. To evaluate the impact of test compounds against these notable pro-angiogenic factors, the gene transcript expression level in chick CAM tissue treated with 1 µM and 10 µM concentrations of NBL, TC, PPR and AXI as standard reference was evaluated using chick RT-PCR primers. A significant decrease in relative gene expression of VEGF-A and VEGFR-2 was observed in the treated tissues in comparison to that of the control and with the most pronounced effect at 10 µM treatment (Fig. 7).

### Antiangiogenic activity of NBL, TC and PPR on CAM U87 xenografts

**Fig. 8:**
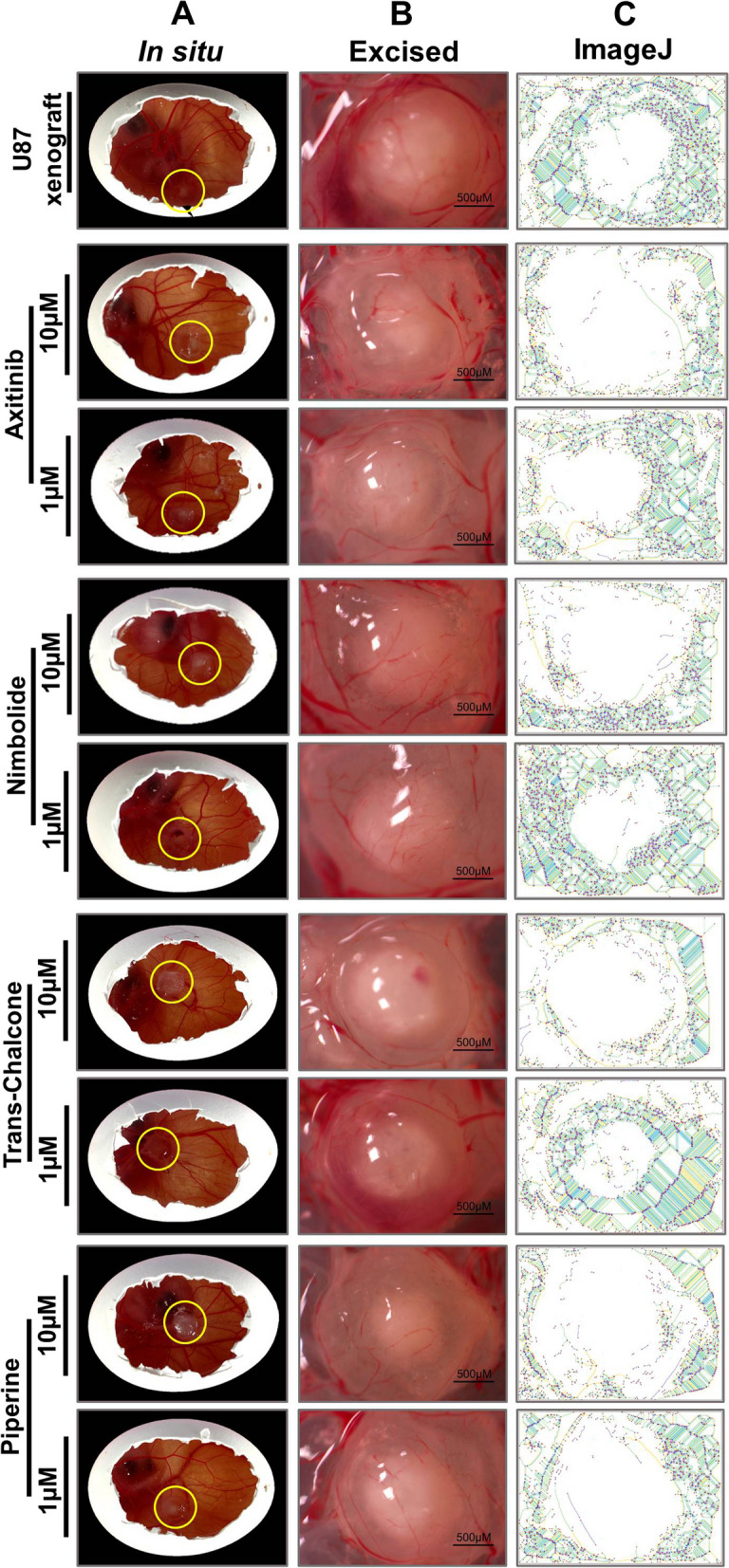

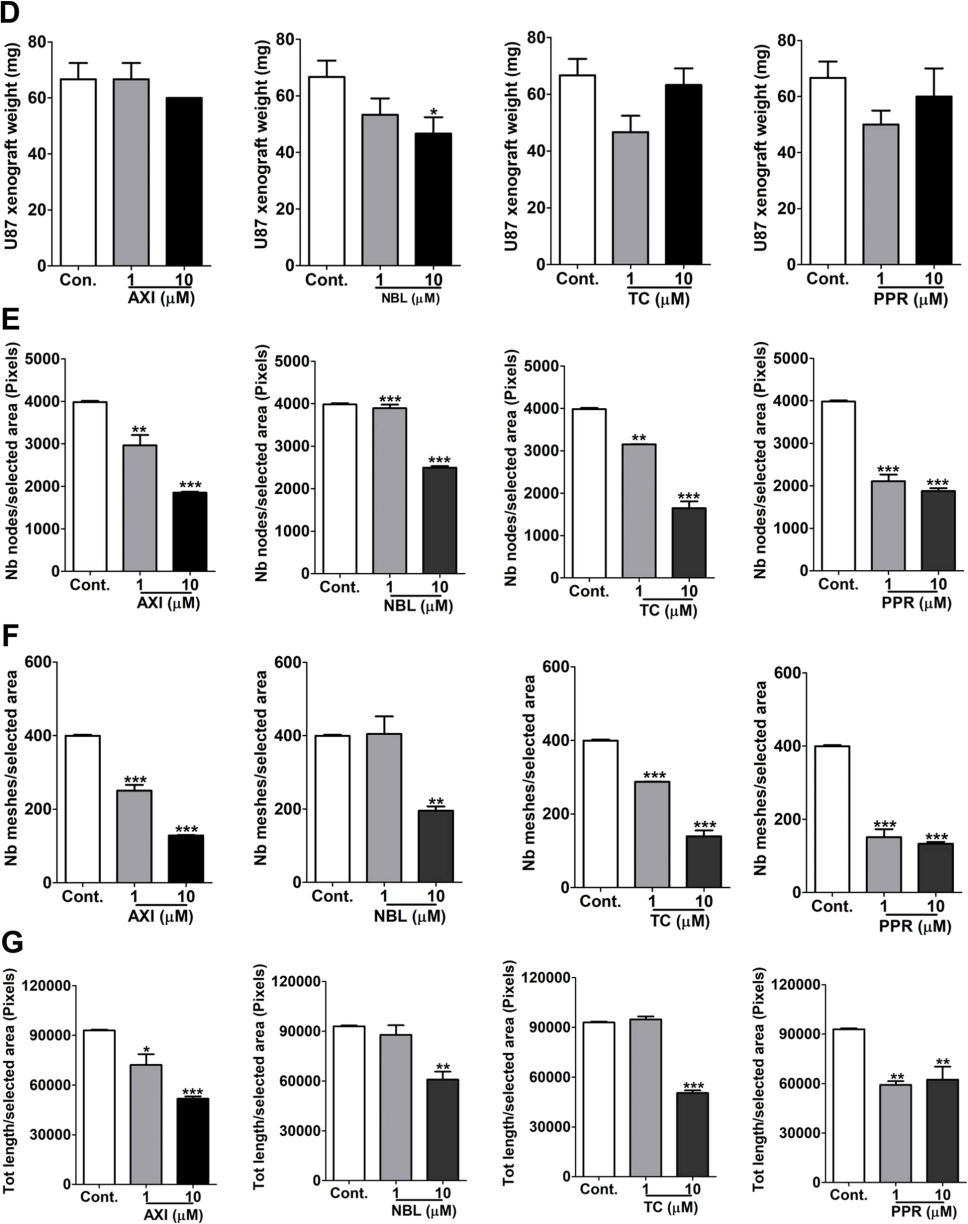
Antiangiogenic effect of AXI, NBL, TC and PPR on chick CAM U87 xenografts generated (EDD9-EDD12). **A-B** In situ images of chick CAM implanted with U87 cells at a density of 1.5 × 10^6^ densities supplemented with different concentrations of treatments and only media for control; **C** Outputs of ImageJ analysis for blood vessel density corresponding in situ (horizontally) image (column B); **D** U87 xenograft volume obtained from different treatments compared with control; **E**–**G** Number of nodes, meshes and total blood vessel length. Statistically significant decreases in parameters representing blood vessel density were observed in the treated compared to the control. *, **, *** = significant with *p*-value < 0.05, < 0.001 & < 0.0001 respectively

Having observed the test compounds to effectively inhibit blood vessel density at 1 and 10 µM, as well as reduced VEGF-A and VEGFR-2 gene transcript expression in CAM, the effect was further evaluated in CAM U87 xenograft models for GBM-induced angiogenesis. The tumor cells supplemented with the test compounds were implanted onto the CAM on EDD9. Seventy-two hours post tumor cell implantation, on EDD13 the xenografts were excised, weighted, and quantitatively evaluated for CAM vasculature at the tumor site by ImageJ. No significant difference in tumor weight was observed between the control and treatments except for NBL at 10 µM. The xenografts treated with 10 µM NBL exhibited the most pronounced reduction in tumor weight. Quantitative evaluation of blood vascular density on CAM at the site of U87 cell implantation or U87 xenografts with ImageJ showed a significant reduction in the nodes, meshes and total length in CAM vasculature in comparison to control (Fig. 8).

### Blood vascular density determination in CAM U87 xenograft

**Fig. 9:**
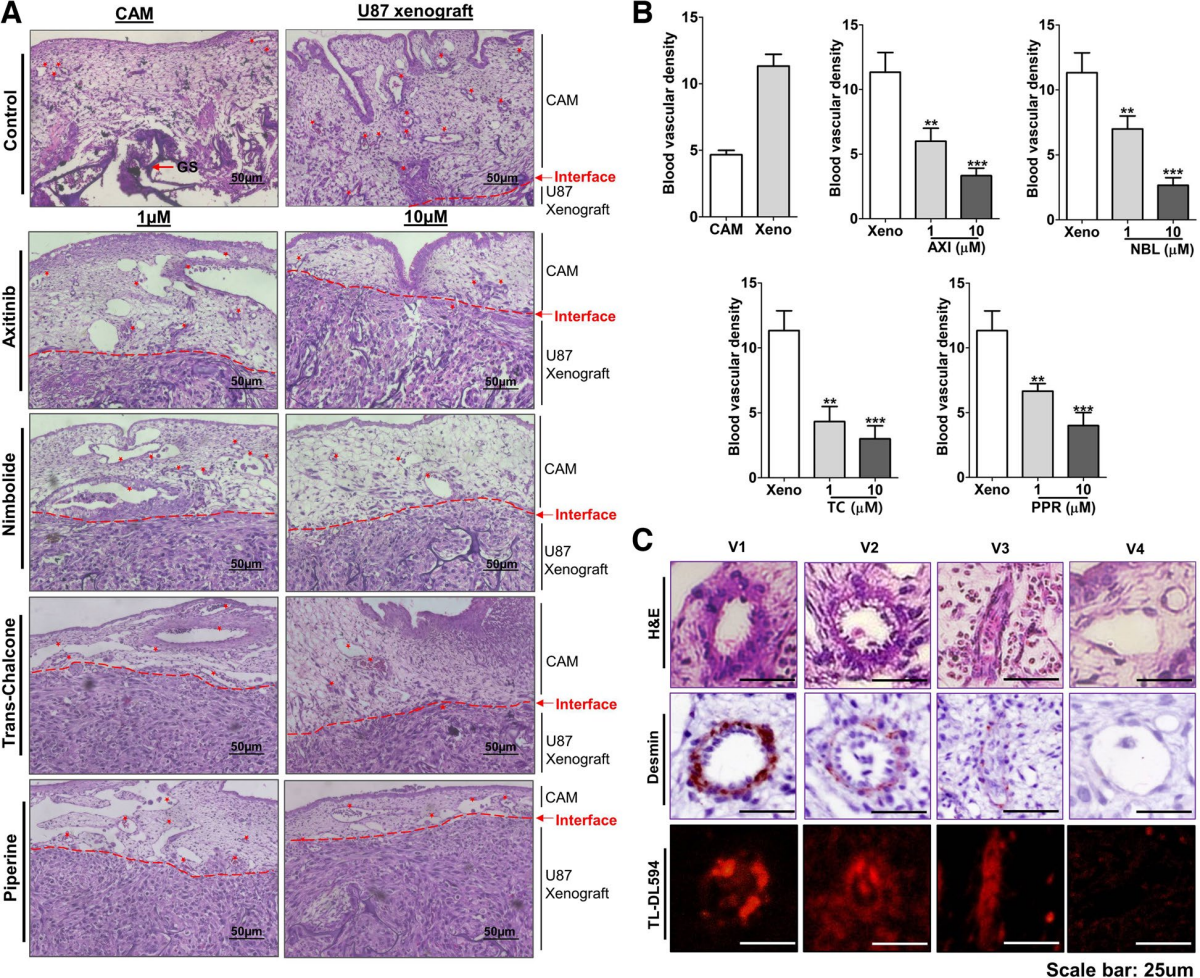
Blood vascular density determination in CAM U87 xenograft. **A** Photographs of H&E staining showing demarcation of CAM and xenograft tissues across all treatments; **B** Graphical representation of blood vessel density across all treatments; **C** Blood vessel showing different histomorphology staining: H&E stained (upper row), labelled with antibodies against desmin (middle row), labelled with lectin (lower row). **, *** = significant with *p*-value < 0.001 & < 0.0001 respectively. Abbreviations: V-vessel; TL-DL594-Tomato lectin DyLight 594

The xenografts of treated as well as non-treated U87 cells were evaluated for blood vessel density within the tumor xenografts demarcated with histochemistry. The H&E staining reveals the blood vessels to be more concentrated around the CAM-xenograft interface (Fig. 9A-B). The blood vessels were identified by the presence of a defined boundary and a lumen with or without blood cells. An increase in blood vessel density was observed in tumor xenografts with no treatment compared to negative control representing tumor-induced angiogenesis. The histological sections of CAM with either AXI, NBL, TC or PPR treated xenografts indicate a significant decrease in blood vessel density compared to control U87 bearing CAM, in a dose-dependent manner. TC showed a strong inhibitory response at 1 µM, however, at 10 µM all the agents showed comparable inhibition ranging from 60–70% w.r.t density recorded in control U87 xenografts. The blood vessels identified with H&E staining corresponded with those indicated by TL histochemistry (red fluorescence) and desmin IHC represented by the presence of a brownish reaction product (Fig. 9C).

### Effect of NBL, TC and PPR on VEGF-A and VEGFR-2 transcript in CAM U87 xenograft

**Fig. 10:**
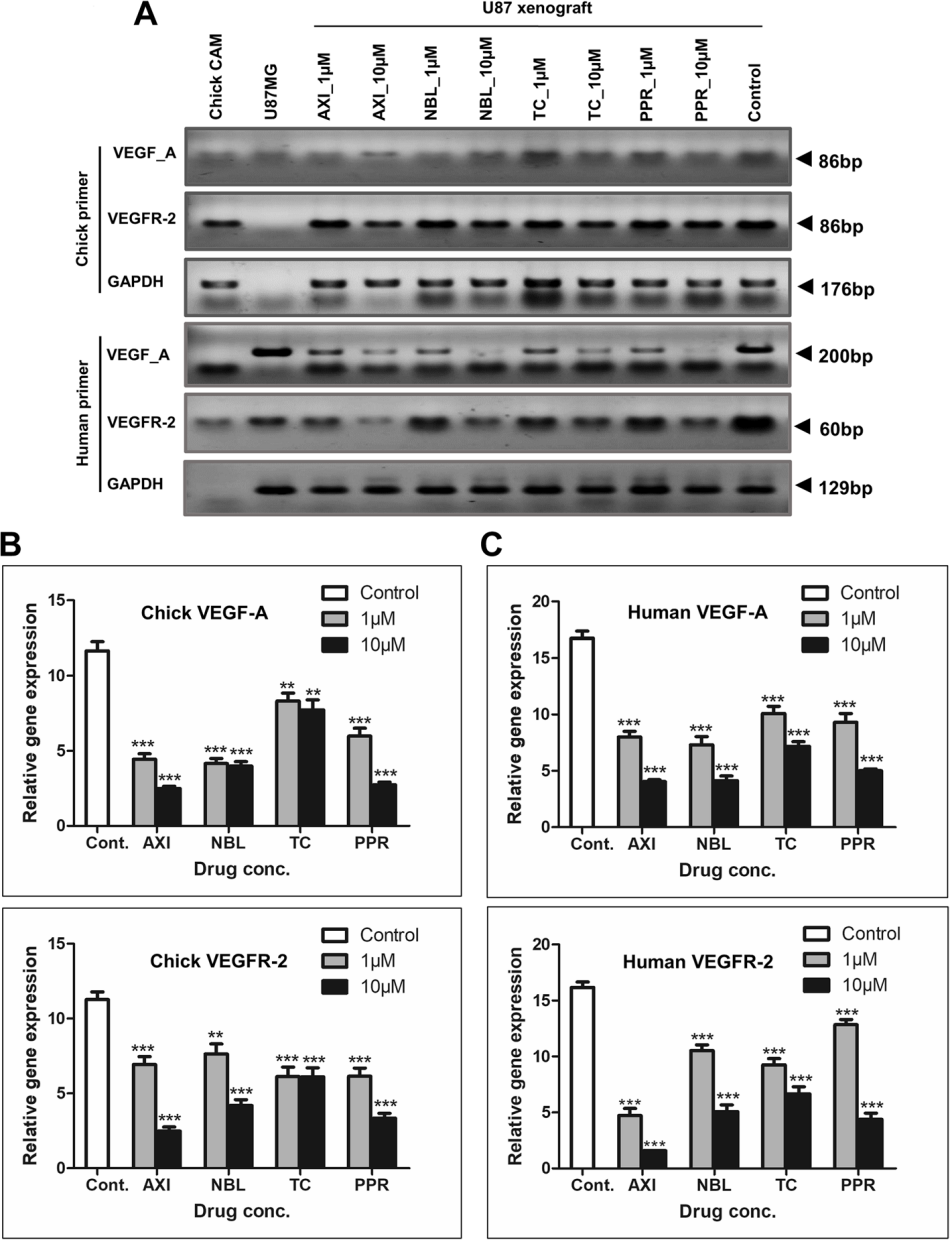
Assessment of angiogenesis related transcripts in CAM U87 xenografts. **A** Gel images showing transcript expression of VEGF-A and VEGFR-2 in chick CAM U87 xenograft (Full gel images included in Supplementary Information File); **B** & **C** Densitometric evaluation of chick and human VEGF-A & VEGFR-2 gene level expression in CAM U87 xenografts normalized to GAPDH transcript expression. **, *** = significant with *p*-value < 0.001 & < 0.0001 respectively

CAM U87 xenografts are composed of chick and human tissue therefore dissection of human versus chick contribution in xenograft angiogenesis and the influence of AXI, NBL, TC and PPR on respected VEGF-A and VEGFR-2 gene transcript expression were evaluated for both chicks and human origin using specific primers (Fig. 10). In RT-PCR performed using specific primers for chick VEGF-A and VEGFR-2 on RNA derived from chick CAM only, distinct bands of VEGF-A and VEGFR-2 were observed. Incidentally, RT-PCR with these primers on U87 RNA showed the same cross-reactivity of the chick VEGF-A primers with human U87 transcripts but no cross-reactivity was observed between chick VEGFR-2 and human transcripts. Similarly, human VEGF-A and VEGFR-2 primers specifically amplified corresponding human transcripts, however, only a minor cross-reactivity of human VEGFR-2 primers was detected for chick transcripts. In CAM U87 xenograft (positive control), distinct amplification bands were observed for both chick and human VEGF-A and VEGFR-2 transcripts, however, CAM U87 xenografts treated with different drugs at indicated concentrations (1 & 10 µM) displayed variable degrees of inhibition as compared to the control in a dose-dependent manner. AXI which was a positive reference drug effectively blocked chick and human VEGF-A and VEGFR-2 at both the concentrations tested (1 and 10 µM) dose-dependent manner. A similar pattern of effective blockage of both chick VEGF-A and VEGFR-2 transcript was observed with NBL, TC and PPR. Amongst the test compounds, TC exhibited the least effect on VEGF-A transcript inhibition. Whereas NBL at 1 µM showed the least inhibitory effect on VEGFR-2 transcript compared to AXI, TC and PPR. Human VEGF-A and VEGFR-2 transcript analysis showed effective inhibition of human VEGF-A and VEGFR-2 transcripts at the test concentrations by all the test compounds, AXI, NBL, TC and PPR compared to negative control. Notably, PPR showed a strong dose-dependent effect on human and chick VEGF transcript and was equally effective in preventing the chick VEGFR-2 transcript expression. Similarly, TC was antiangiogenic with a more pronounced effect on human VEGF transcripts and chick VEGFR-2 expression.

## Discussion

The phytochemicals NBL, TC, PPR have reported anti-angiogenic activity in several cancer or other disease conditions, however, no study has reported the antiangiogenic property of these phytochemicals against key angiogenic factors VEGFA/VEGFR-2 axis in GBM nor in GBM xenograft CAM model. So, for the first time we have carried out a comparative analysis of anti-angiogenic activity of these phytochemicals in a single model system to find the most potent lead. We evaluated the antiangiogenic properties of NBL, TC and PPR in an in vivo chick CAM angiogenesis model with AXI as the reference drug. Using in vitro 2D and 3D U87 GBM models, the non-cytotoxic dose of test compounds was determined to be < 11 µM for in vivo CAM study. The phytochemicals showed a significant decrease in blood vascular density on CAM angiogenesis as well as histological sections of CAM U87 xenograft. Significant decrease in VEGF-A and VEGFR-2 transcript level expression in NBL, TC, and PPR treated CAM as a stand-alone as well as CAM U87 xenografts. Our study indicates that NBL, TC, and PPR have the potential to inhibit angiogenesis in the GBM micro-environment through VEGFR-2 in ECs thus preventing neo-angiogenesis.

To avoid confounding evaluation of the anti-cancer property as antiangiogenic property, the effect of NBL, TC, and PPR was evaluated on U87 cells in different culture conditions namely 2D monolayer and 3D spheroids. The number of healthy or viable U87 cells corresponding to the susceptibility of U87 cells to different concentrations of AXI, NBL, TC and PPR were determined by MTT colorimetric assay. MTT assay is one of the most versatile cell viability assays that rely on the conversion of substrate MTT [3-(4,5-dimethylthiazol-2- yl)-2,5-diphenyltetrazolium bromide] to the chromogenic product by the action of mitochondrial reductase in live cells [37]. Mitochondrial activity directly corresponds to the number of viable cells in culture. This is a routine assay in pharmacology and toxicology. All the drugs tested showed strong dose-dependent loss of cell viability at concentrations ≥ IC50 compared to the control, however, they also provided probable working range to avoid drug-induced toxicities. Nevertheless, drug sensitivities drastically change with methods of cell culture and drugs tend to become ineffective when cells move from 2 to 3D spheroid cultures [38]. The latter remains to be a clearer recapitulation of the in vivo situation and behaviour of the tumor tissue.

In continuation to U87 viability evaluated in a 2D culture system, the viability of U87 as a 3D in vivo GBM model on CAM was evaluated by the generation of U87 3D spheroids incubated in culture media supplemented with different concentrations of AXI, NBL, TC and PPR using HD and LOT methods. Firstly, the ability of U87 cells to spontaneously form 3D spheroids was evaluated. U87 cells consistently formed definite spheroids within 48–96 h post incubation as seen in earlier reports [39] when provided with an optimal environment for aggregation such as HD [40] and LOT using 96-well ultra-low attachment (ULA) plate [41] methods of spheroid generation. The majority of the cell lines do not form spheroids in normal 2D monoculture, however, in the case of U87 cell lines, spheroids were observed routinely even in monolayer culture at a high density which corroborated with other studies [42]. It appears to be an inherent property of U87. 3D tumor spheroids are one of the most characterized in vitro cancer model systems having a close resemblance to the patho-physiological tissue organization and complexity bearing cell–cell or cell–matrix interactions [43, 44]. It has several applications in biomedical research with cell viability assessment being one aspect which could be easily evaluated through bright-field microscopy [45]. The effect of efficient and effective drug delivery of niosomal formulations containing curcumin and doxorubicin on U87 3D spheroids exhibited reduced spheroid size/diameter, distorted spheroidal shape, and lack of tight compact aggregation [26]. Similar morphological alterations of reduced spheroid size/volume, loss of spheroid integrity, or loosely dispersed cells were observed in U87 3D spheroids treated with higher test compound concentration but the absence of such features in spheroids treated with lower drug concentration i.e., < 11 µM indicate reduced or lack of cytotoxic effect on U87 cells at these concentrations.

The morphometric alterations in response to drug treatment or cell death were further validated by dead cell stains, PI and DAPI [46, 47]. PI intercalates between double-stranded DNA base pairs, excited between 400–600 nm and emits light between 600–700 nm wavelengths [48]. DAPI on entering the cell membrane binds with DNA and forms a DAPI-DNA complex exhibiting blue fluorescence (wavelength of excitation and emission of the complex = 360 nm and 460 nm, respectively) [49, 50]. The U87 cell viability in 3D spheroids determined by altered morphological appearance strongly corroborated with fluorescence emission increasing in higher treatment concentrations. However, there was no such signature at a low dose less than 11 µM. Using U87 3D spheroids, NBL, TC and PPR showed no recorded loss of U87 cell viability or spheroid morphology at concentrations ≤ 11 µM. Therefore, these observations confirmed that any change in tumor characteristics in CAM xenografts due to antiangiogenic drugs could be directly attributed to their antiangiogenic property.

The Chick CAM model is a widely used in vivo angiogenic model, densely vascularized, easy to manipulate and macroscopic. Using CAM models, the antiangiogenic effect of test compounds can be conveniently determined by a decrease in blood vascular density, branch points, length, CAM area and vascular disorganization at the experimental site on CAM [51–54]. In the present study, NBL, TC, PPR (at 1 & 10 µM) and AXI (at 1 µM) showed these characteristic features representing the antiangiogenic effect of these agents. Similar results have been shown with *Usena antarctica* [55] and chebulinic acid [53] on the CAM.

VEGFR-2 is the main receptor for VEGF-A, the principle pro-angiogenic factor. The activation of VEGFR-2 on VEGF-A binding promotes vital angiogenesis processes such as EC proliferation, migration, and survival. During both physiological and pathological angiogenesis, the level of VEGFR-2 is generally high [56]. Suppressed VEGFR-2 results in early embryonic death and deformities of endothelial and hematopoietic cells [57]. Our study on CAM as a stand-alone angiogenesis model with test compounds AXI, NBL, TC, and PPR for their antiangiogenic activity showed a decrease in both VEGF-A and VEGFR-2 transcript levels indicating downregulation of angiogenic processes. Having found the test compounds AXI, NBL, TC, and PPR to show effective antiangiogenic activity such as suppressed vascular density as well as reduced VEGF-A and VEGFR-2 transcript level, the study was further taken to the next stage for evaluation using CAM U87 xenografts.

Tumor bearing chick CAM models serve as a suitable cost-effective alternative to tumor bearing higher murine models for the evaluation of novel antiangiogenic compounds against tumor-induced angiogenesis. Phytochemicals such as berbamine [58] and tetrandrine [59] were reported to inhibit angiogenesis through chick CAM bearing glioblastoma U87 cells which exhibited reduced neovascularization, capillaries, vascular or CAM area. Several other phytochemicals such as myricetin [60], galangin [60], theasaponin E1 [61], and theaflavin-3, 3'-digallate [62] have also been reported to possess antiangiogenic activity and suppressed blood vessel density in CAM bearing serous ovarian adenocarcinoma OVCAR-3 cell line. These studies strongly support our findings of suppressed blood vascular density in the U87 bearing chick CAM model when supplemented with NBL, TC and PPR.

Evaluation of vascular density in tumor xenografts has been commonly used for the determination of angiogenesis [63, 64] or the evaluation of the antiangiogenic activity of novel compounds [65]. Blood vessels could be easily identified by their morphology from histological sections using H&E staining [66]. Our study showed an increased vascular network in the CAM bearing control U87 cells compared to the negative control (CAM only), while, treated U87 CAM xenografts showed decreased vascular network in a dose-dependent manner compared to the positive control (U87 bearing CAM without treatment). The antiangiogenic activity of heterobimetallic Ruthenium complexes has been evaluated by morphological features in CAM using H&E staining [67]. The variability and identification of blood vessels in H&E-stained U87 CAM xenograft sections were validated by TL [68] and desmin [69]. TL is an effective blood vessel marker with a strong affinity to polylactosamine oligosaccharides present on ECs as well as epithelial cell surfaces. Desmin is an intermediate filament protein localised in EC, smooth and skeletal muscles as well as a marker for pericytes associated with blood vessels throughout angiogenesis [70]. Further, to ascertain the role of notable angiogenic factors, VEGF-A and VEGFR-2 in the suppression of angiogenic activity in CAM U87 xenograft by the test compounds, their gene transcript level expression was determined.

VEGFR-2 plays a critical role in vascular system development/angiogenesis. It is often upregulated in solid tumors whose growth is dependent on nutrients obtained through blood supply [71, 72]. Our study showed decreased gene transcript expression of VEGFR-2 and VEGF-A in CAM U87 xenografts treated with AXI, NBL, TC and PPR. The consistent exhibition of suppressed expression of VEGF-A and VEGFR-2 in CAM as a stand-alone angiogenic model as well as in CAM U87 xenograft in response to test compounds (AXI, NBL, TC and PPR) affirms the antiangiogenic activity of NBL, TC and PPR. Additionally, AXI is an FDA-approved antiangiogenic compound with the property to inhibit VEGFR-2 activity [73] thus strengthening our observations of the antiangiogenic activity of these phytochemicals. Several studies have reported decreased VEGF-A/VEGFR-2 expression in tumor xenografts in response to antiangiogenic agents. Human melanoma A375 cell bearing immunodeficient mice exhibited reduced VEGF-A/VEGFR-2 expression in response to the antiangiogenic activity of rapamycin [74]. A similar response of decreased VEGF-A/VEGFR-2 expression has been shown in U87 GBM cell bearing chick CAM to berbamine [58].

Taken altogether, these findings suggest that NBL, TC and PPR could be used as novel inhibitors of the VEGF/VEGFR axis in the treatment of GBM. Amongst these phytochemicals, PPR was found to be most effective inhibitor of human VEGF-A and chick VEGFR-2, the key angiogenic stimulators in CAM U87 xenograft angiogenesis in our study.

### Supplementary Information

**Additional file 1.** 

## Data Availability

The data sets used and or/analyzed during the current study are available from the corresponding author upon reasonable request.

## Abbreviations

2D: 2-Dimensional
3D: 3-Dimensional
AXI: Axitinib
bFGF: Basic fibroblast growth factor
BBB: Blood–brain barrier
CAM: Chorioallantoic membrane
CNS: Central nervous system
cDNA: Complementary DNA
EC: Endothelial cell
EDD: Embryonic development day
GBM: Glioblastoma multiforme
GS: Gelatin sponge
HH Stages: Hamburger–Hamilton stages
HD: Hanging drop
H&E: Haematoxylin and eosin
IHC: Immunohistochemistry
LOT: Liquid overlay technique
NBL: Nimbolide
PCR: Polymerase chain reaction
PPR: Piperine
TC: Trans-chalcone
TL-DL594: Tomato lectin DyLight 594
RT-PCR: Reverse transcriptase–polymerase chain reaction
VEGF-A: Vascular endothelial growth factor A
VEGFR-2: Vascular endothelial growth factors receptor 2

## References

[1] Ostrom QT (2020). CBTRUS Statistical Report: Primary Brain and Other Central Nervous System Tumors Diagnosed in the United States in 2013–2017. Neuro Oncol.

[2] Tamimi AF, Juweid M. Epidemiology and Outcome of Glioblastoma. In: De Vleeschouwer S, editor. Glioblastoma [Internet]. Brisbane: Codon Publications; 2017. Chapter 8. Available from: https://www.ncbi.nlm.nih.gov/books/NBK470003/. 10.15586/codon.glioblastoma.2017.ch8. 29251870

[3] Belghali MY (2021). Epidemiological, clinical, therapeutic and evolutionary features of patients with glioblastoma: series of cases managed in the Department of Hematology-Oncology at the Mohammed VI University Hospital Center in Marrakech in 2016 and 2017. Pan Afr Med J.

[4] Alphandery E (2018). Glioblastoma treatments: an account of recent industrial developments. Front Pharmacol.

[5] WHO. World: Brain, Central Nervous System. 2020. Available from: https://gco.iarc.fr/today/data/factsheets/populations/900-world-fact-sheets.pdf. Cited 2023 6th May 2023

[6] Leece R (2017). Global incidence of malignant brain and other central nervous system tumors by histology, 2003–2007. Neuro Oncol.

[7] Johnson DR, O'Neill BP (2012). Glioblastoma survival in the United States before and during the temozolomide era. J Neurooncol.

[8] Daubon T (2020). Glioblastoma Immune Landscape and the Potential of New Immunotherapies. Front Immunol.

[9] Fischer I (2005). Angiogenesis in gliomas: biology and molecular pathophysiology. Brain Pathol.

[10] Wurdinger T, Tannous BA (2009). Glioma angiogenesis: Towards novel RNA therapeutics. Cell Adh Migr.

[11] Ahir BK, Engelhard HH, Lakka SS (2020). Tumor development and angiogenesis in adult brain tumor: glioblastoma. Mol Neurobiol.

[12] Xu C, Wu X, Zhu J (2013). VEGF promotes proliferation of human glioblastoma multiforme stem-like cells through VEGF receptor 2. Sci World J.

[13] Claesson-Welsh L, Welsh M (2013). VEGFA and tumour angiogenesis. J Intern Med.

[14] Zhao Y, Adjei AA (2015). Targeting angiogenesis in cancer therapy: moving beyond vascular endothelial growth factor. Oncologist.

[15] Navis AC (2013). Effects of dual targeting of tumor cells and stroma in human glioblastoma xenografts with a tyrosine kinase inhibitor against c-MET and VEGFR2. PLoS One.

[16] Hamerlik P (2012). Autocrine VEGF-VEGFR2-Neuropilin-1 signaling promotes glioma stem-like cell viability and tumor growth. J Exp Med.

[17] Michaelsen SR (2018). VEGF-C sustains VEGFR2 activation under bevacizumab therapy and promotes glioblastoma maintenance. Neuro Oncol.

[18] Angiogenesis inhibitors. 2018. Available from: https://www.cancer.gov/about-cancer/treatment/types/immunotherapy/angiogenesis-inhibitors-fact-sheet#. Cited 2022 14th June.

[19] Thomson RJ, Moshirfar M, Ronquillo Y. Tyrosine Kinase Inhibitors [Internet]. [Updated 2023 Jul 18]. In: StatPearls. Treasure Island: StatPearls Publishing; 2023. Available from: https://www.ncbi.nlm.nih.gov/books/NBK563322/. 33090752

[20] Jaszai J, Schmidt MHH (2019). Trends and Challenges in Tumor Antiangiogenic Therapies. Cells..

[21] Fontanella C (2014). Clinical advances in the development of novel VEGFR2 inhibitors. Ann Transl Med.

[22] Nishida N (2006). Angiogenesis in cancer. Vasc Health Risk Manag.

[23] Ziyad S, Iruela-Arispe ML (2011). Molecular mechanisms of tumor angiogenesis. Genes Cancer.

[24] Wanigasekara J (2023). Three-Dimensional (3D) in vitro cell culture protocols to enhance glioblastoma research. PLoS ONE.

[25] Foty R. A simple hanging drop cell culture protocol for generation of 3D spheroids. J Vis Exp. 2011;(51):2720. 10.3791/2720 PMC3197119 21587162

[26] Seleci DA (2017). Tumor homing and penetrating peptide-conjugated niosomes as multi-drug carriers for tumor-targeted drug delivery. RSC Adv.

[27] Tomayko MM, Reynolds CP (1989). Determination of subcutaneous tumor size in athymic (nude) mice. Cancer Chemother Pharmacol.

[28] Ribatti D (2006). The gelatin sponge-chorioallantoic membrane assay. Nat Protoc.

[29] Cloney K, Franz-Odendaal TA (2015). Optimized ex-ovo culturing of chick embryos to advanced stages of development. J Vis Exp.

[30] Schneider CA, Rasband WS, Eliceiri KW (2012). NIH Image to ImageJ: 25 years of image analysis. Nat Methods.

[31] Chen J (2015). The flavonoid nobiletin inhibits tumor growth and angiogenesis of ovarian cancers via the Akt pathway. Int J Oncol.

[32] West DC (2001). Angiogenesis assays using chick chorioallantoic membrane. Methods Mol Med.

[33] Feldman AT, Wolfe D (2014). Tissue processing and hematoxylin and eosin staining. Methods Mol Biol.

[34] Villacampa N (2013). Tomato lectin histochemistry for microglial visualization. Methods Mol Biol.

[35] Bhat A, Sharma A, Bharti AC (2018). Upstream Hedgehog signaling components are exported in exosomes of cervical cancer cell lines. Nanomedicine (Lond).

[36] Vishnoi K (2016). Cross-talk between human papillomavirus Oncoproteins and hedgehog signaling synergistically promotes Stemness in cervical cancer cells. Sci Rep.

[37] Kumar P, Nagarajan A, Uchil PD. Analysis of Cell Viability by the MTT Assay. Cold Spring Harb Protoc. 2018;2018(6). 10.1109/CVPR.2018.00745. 10.1101/pdb.prot095505 29858338

[38] Melissaridou S (2019). The effect of 2D and 3D cell cultures on treatment response, EMT profile and stem cell features in head and neck cancer. Cancer Cell Int.

[39] Vinci M (2012). Advances in establishment and analysis of three-dimensional tumor spheroid-based functional assays for target validation and drug evaluation. BMC Biol.

[40] Liu X (2021). A Novel SimpleDrop Chip for 3D Spheroid Formation and Anti-Cancer Drug Assay. Micromachines (Basel)..

[41] Howes AL (2014). 3-Dimensional culture systems for anti-cancer compound profiling and high-throughput screening reveal increases in EGFR inhibitor-mediated cytotoxicity compared to monolayer culture systems. PLoS One.

[42] Diao W (2019). Behaviors of glioblastoma cells in in vitro microenvironments. Sci Rep.

[43] Vinci M (2013). Tumor spheroid-based migration assays for evaluation of therapeutic agents. Methods Mol Biol.

[44] Rolver MG, Elingaard-Larsen LO, Pedersen SF. Assessing Cell Viability and Death in 3D Spheroid Cultures of Cancer Cells. J Vis Exp. 2019;(148). 10.3791/59714. 10.3791/59714 31259899

[45] Gebhard C, Gabriel C, Walter I (2016). Morphological and immunohistochemical characterization of canine osteosarcoma spheroid cell cultures. Anat Histol Embryol.

[46] Wallberg F, Tenev T, Meier P (2016). Analysis of apoptosis and necroptosis by fluorescence-activated cell sorting. Cold Spring Harb Protoc.

[47] Crowley LC, Marfell BJ, Waterhouse NJ. Analyzing Cell Death by Nuclear Staining with Hoechst 33342. Cold Spring Harb Protoc. 2016;2016(9). 10.1101/pdb.prot087205. 10.1101/pdb.prot087205 27587774

[48] Crowley LC, Scott AP, Marfell BJ, Boughaba JA, Chojnowski G, Waterhouse NJ. Measuring Cell Death by Propidium Iodide Uptake and Flow Cytometry. Cold Spring Harb Protoc. 2016;2016(7). 10.1101/pdb.prot087163. 10.1101/pdb.prot087163 27371595

[49] Chen H (2017). Clinical significance of ALDH1 combined with DAPI expression in patients with esophageal carcinoma. Oncol Lett.

[50] Otto FJ (1994). High-resolution analysis of nuclear DNA employing the fluorochrome DAPI. Methods Cell Biol.

[51] Ma Q, Chen W, Chen W (2016). Anti-tumor angiogenesis effect of a new compound: B-9-3 through interference with VEGFR2 signaling. Tumour Biol.

[52] Ribatti D (2016). The chick embryo chorioallantoic membrane (CAM). A multifaceted experimental model. Mech Dev.

[53] Shanmuganathan S, Angayarkanni N (2018). Chebulagic acid Chebulinic acid and Gallic acid, the active principles of Triphala, inhibit TNFalpha induced pro-angiogenic and pro-inflammatory activities in retinal capillary endothelial cells by inhibiting p38, ERK and NFkB phosphorylation. Vascul Pharmacol.

[54] Tufan AC, Satiroglu-Tufan NL (2005). The chick embryo chorioallantoic membrane as a model system for the study of tumor angiogenesis, invasion and development of antiangiogenic agents. Curr Cancer Drug Targets.

[55] Petrova K (2022). Usnic Acid Isolated from Usnea antarctica (Du Rietz) Reduced In Vitro Angiogenesis in VEGF- and bFGF-Stimulated HUVECs and Ex Ovo in Quail Chorioallantoic Membrane (CAM) Assay. Life (Basel)..

[56] Wang L, Chen N, Cheng H (2020). Fisetin inhibits vascular endothelial growth factor-induced angiogenesis in retinoblastoma cells. Oncol Lett.

[57] Shalaby F (1995). Failure of blood-island formation and vasculogenesis in Flk-1-deficient mice. Nature.

[58] Kim YJ, Han JM, Jung HJ (2021). Antiangiogenic and antitumor potential of berbamine, a natural CaMKIIgamma inhibitor, against glioblastoma. Biochem Biophys Res Commun.

[59] Ma JW (2015). Tetrandrine suppresses human glioma growth by inhibiting cell survival, proliferation and tumour angiogenesis through attenuating STAT3 phosphorylation. Eur J Pharmacol.

[60] Huang H (2015). Dietary compounds galangin and myricetin suppress ovarian cancer cell angiogenesis. J Funct Foods.

[61] Li B (2021). Theasaponin E1 Inhibits Platinum-Resistant Ovarian Cancer Cells through Activating Apoptosis and Suppressing Angiogenesis. Molecules..

[62] Gao Y (2016). Theaflavin-3, 3'-digallate decreases human ovarian carcinoma OVCAR-3 cell-induced angiogenesis via Akt and Notch-1 pathways, not via MAPK pathways. Int J Oncol.

[63] Foote RL (2005). Evaluation of tumor angiogenesis measured with microvessel density (MVD) as a prognostic indicator in nasopharyngeal carcinoma: results of RTOG 9505. Int J Radiat Oncol Biol Phys.

[64] Saponaro C (2013). VEGF, HIF-1alpha expression and MVD as an angiogenic network in familial breast cancer. PLoS ONE.

[65] da Costa PM (2015). Improvement of in vivo anticancer and antiangiogenic potential of thalidomide derivatives. Chem Biol Interact.

[66] Basu P (2010). Blood flow interplays with elastin: collagen and MMP: TIMP ratios to maintain healthy vascular structure and function. Vasc Health Risk Manag.

[67] Guedes APM (2020). Heterobimetallic Ru(ii)/Fe(ii) complexes as potent anticancer agents against breast cancer cells, inducing apoptosis through multiple targets. Metallomics.

[68] Jilani SM (2003). Selective binding of lectins to embryonic chicken vasculature. J Histochem Cytochem.

[69] Nico B (2004). Desmin-positive pericytes in the chick embryo chorioallantoic membrane in response to fibroblast growth factor-2. Microvasc Res.

[70] Mittal B (2020). Desmin dysregulation in gall bladder cancer. Indian J Med Res.

[71] Majidpoor J, Mortezaee K (2021). Angiogenesis as a hallmark of solid tumors - clinical perspectives. Cell Oncol (Dordr).

[72] Modi SJ, Kulkarni VM (2019). Vascular endothelial growth factor receptor (VEGFR-2)/KDR inhibitors: medicinal chemistry perspective. Med Drug Discov.

[73] Rossler J (2011). The selective VEGFR1-3 inhibitor axitinib (AG-013736) shows antitumor activity in human neuroblastoma xenografts. Int J Cancer.

[74] Wang M (2019). Rapamycin suppresses angiogenesis and lymphangiogenesis in melanoma by downregulating VEGF-A/VEGFR-2 and VEGF-C/VEGFR-3 expression. Onco Targets Ther.

