# Supplementary material for: In vivo antiangiogenic effect of nimbolide, trans-chalcone and piperine for use against glioblastoma
Source: BMC Cancer. 2023 Nov 30;23:1173. doi: 10.1186/s12885-023-11625-4 (PMC10691152; doi:10.1186/s12885-023-11625-4)
Supplement: Supplementary file 1 — Additional file 1. [file 12885_2023_11625_MOESM1_ESM.zip › SF_Gel Images _PDF_ACB_20231005.pdf]

Chick CAM standalone tissue: VEGF-A chick primer

Figure 7A (Representative Blot), Replicate 1

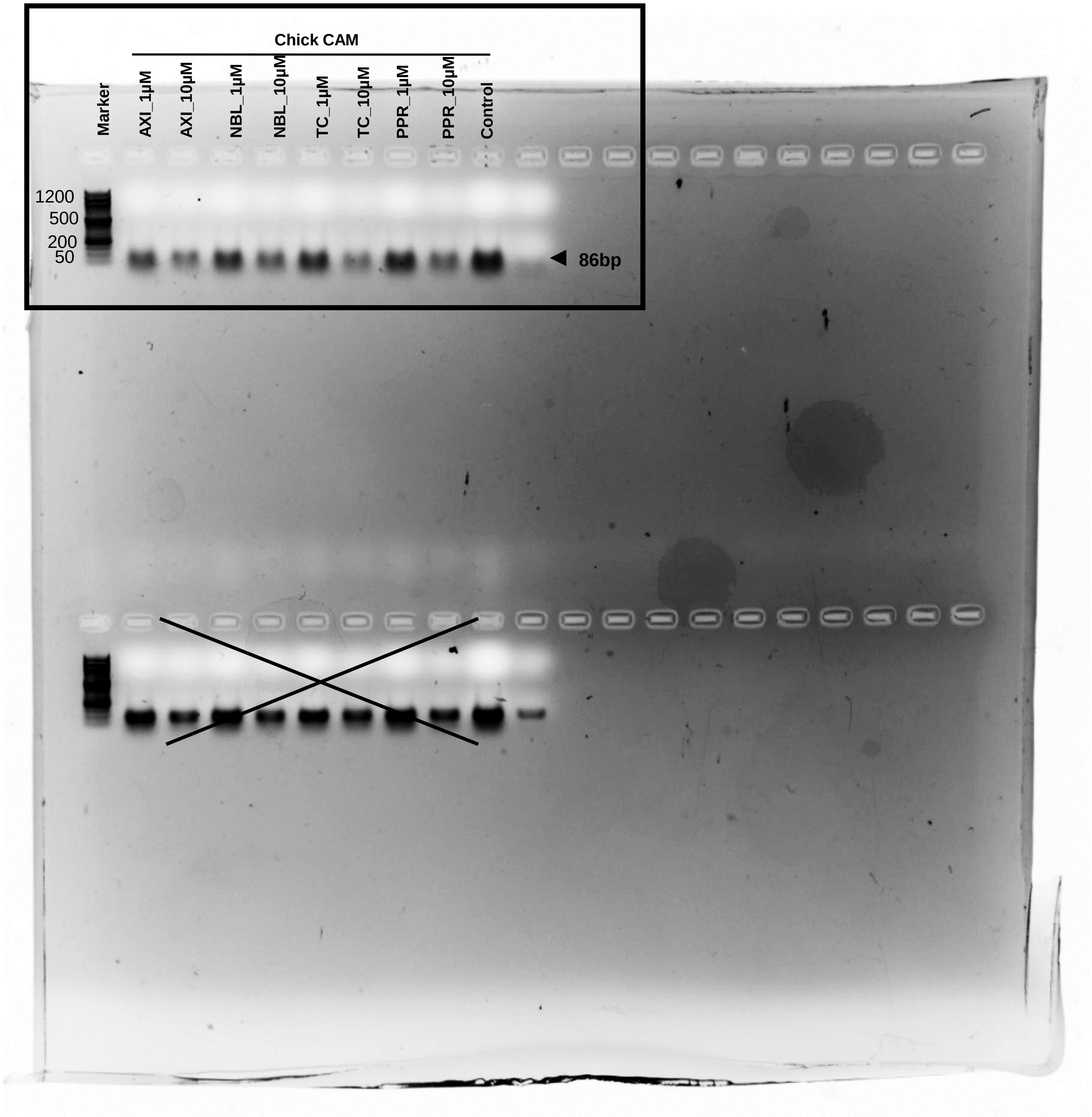

Black box – Representative gel image incorporated in the text

Chick CAM standalone tissue: VEGF-A chick primer

Replicate 2

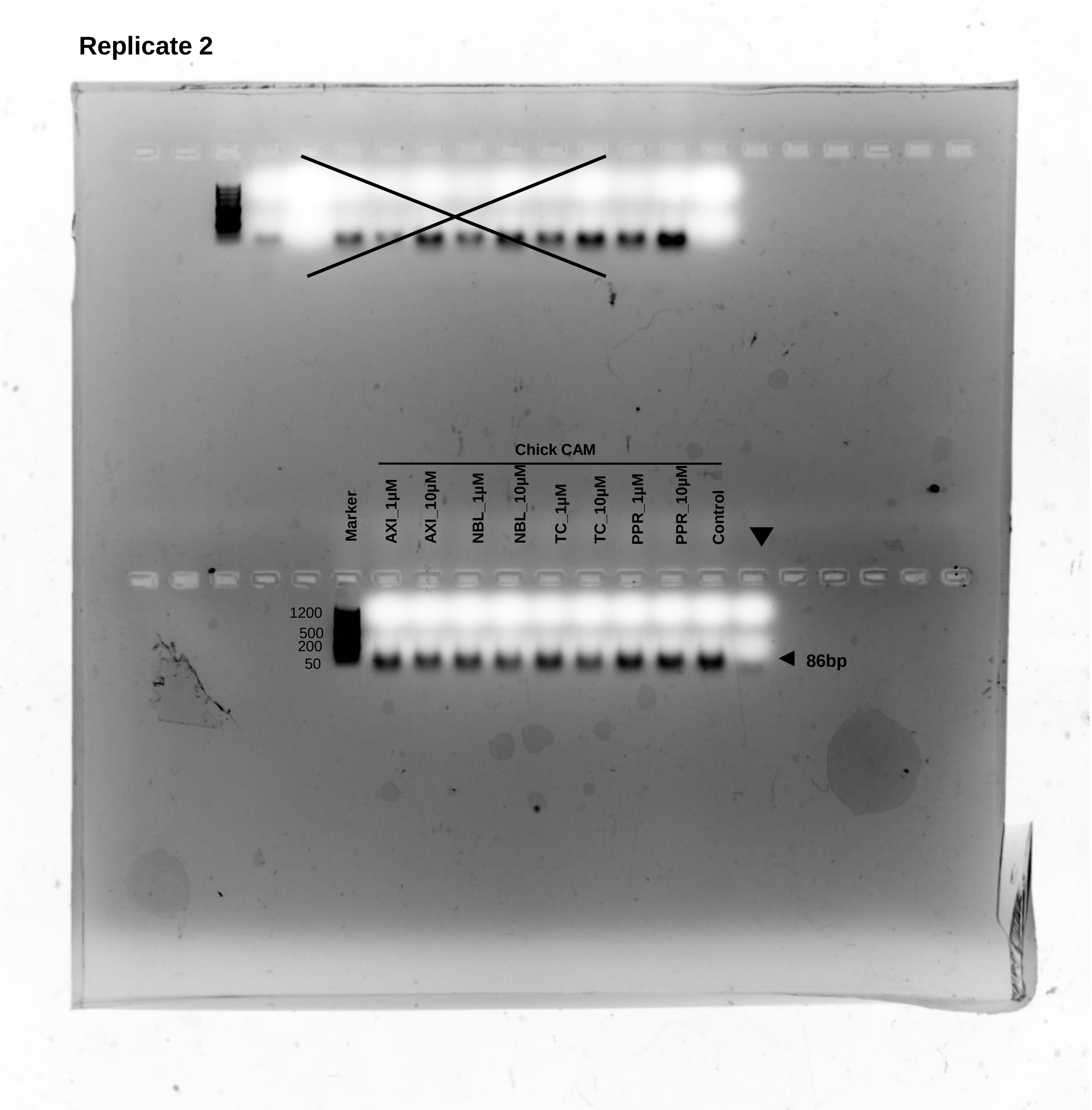

Chick CAM standalone tissue: VEGF-A chick primer

Replicate 3

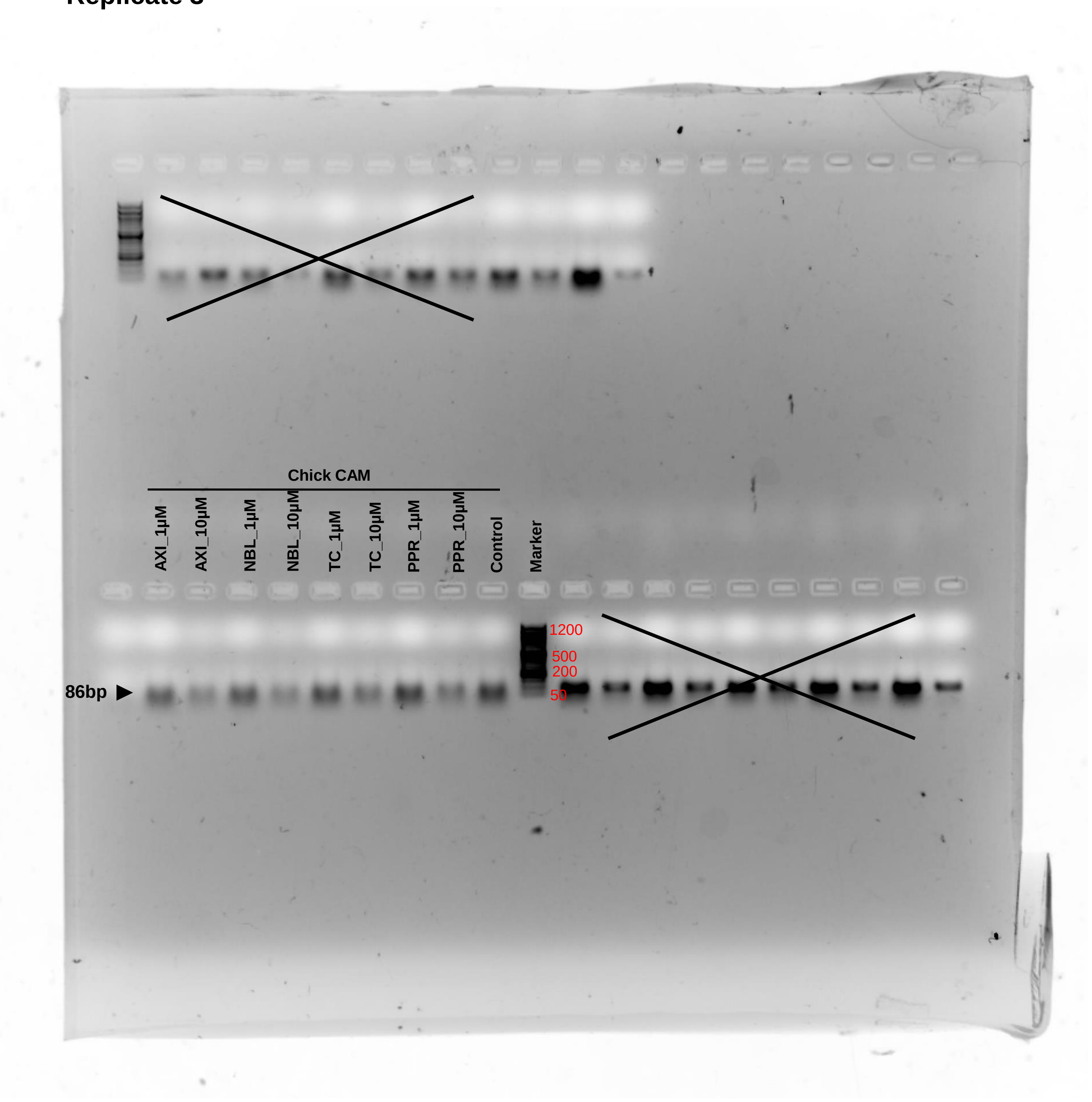

Chick CAM standalone tissue: VEGFR-2 chick primer

Figure 7A (Representative Blot), Replicate 1

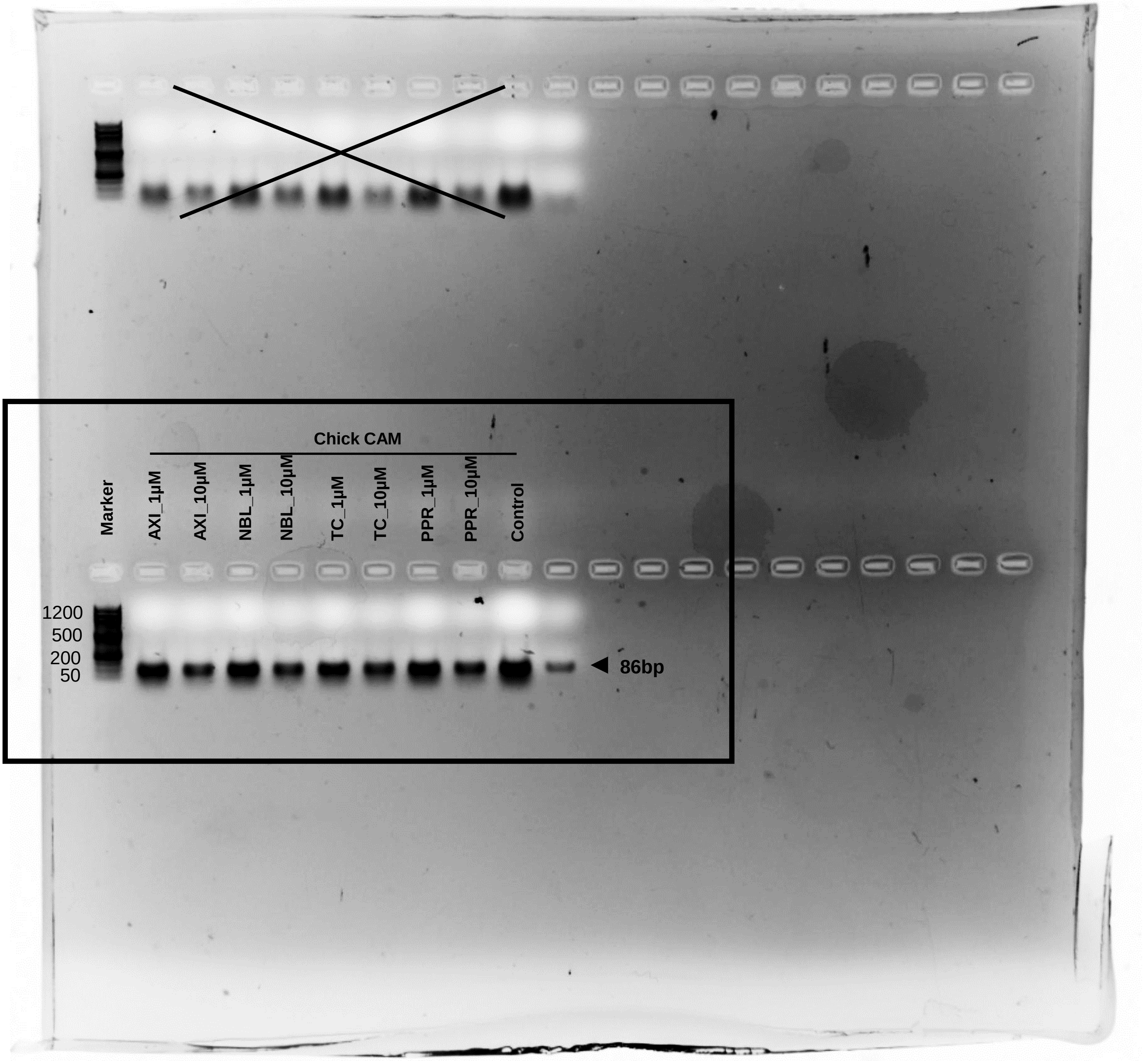

Black box – Representative gel image incorporated in the text

Chick CAM standalone tissue: VEGFR-2 chick primer

Replicate 2

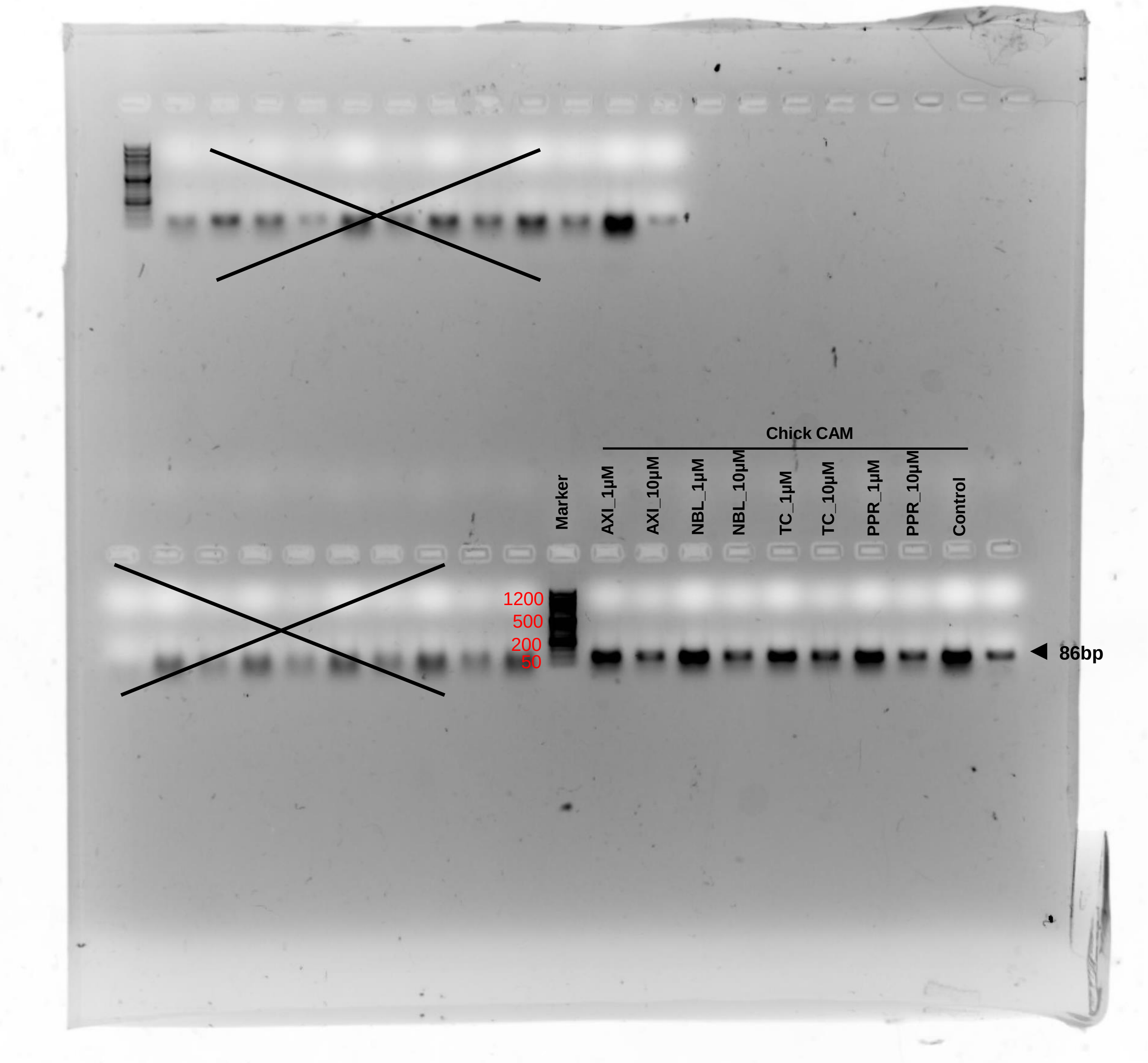

Chick CAM standalone tissue: VEGFR-2 chick primer

Replicate 3

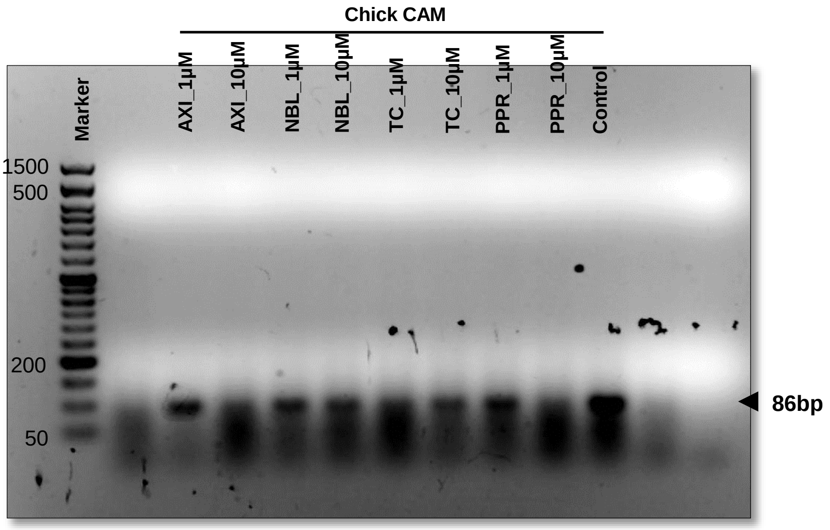

Chick CAM standalone tissue: GAPDH chick primer

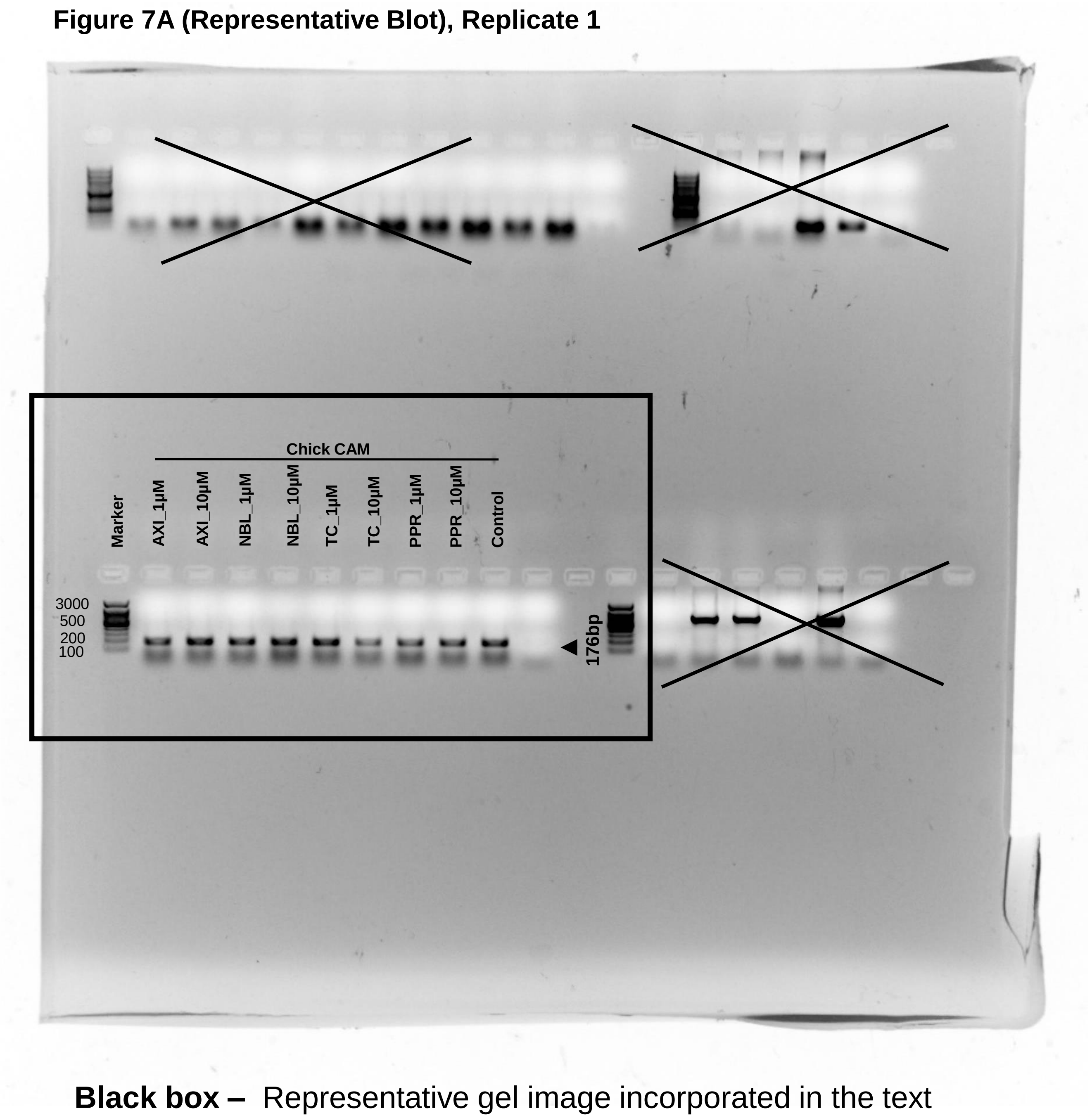

Black box – Representative gel image incorporated in the text

Chick CAM standalone tissue: GAPDH chick primer

Replicate 2

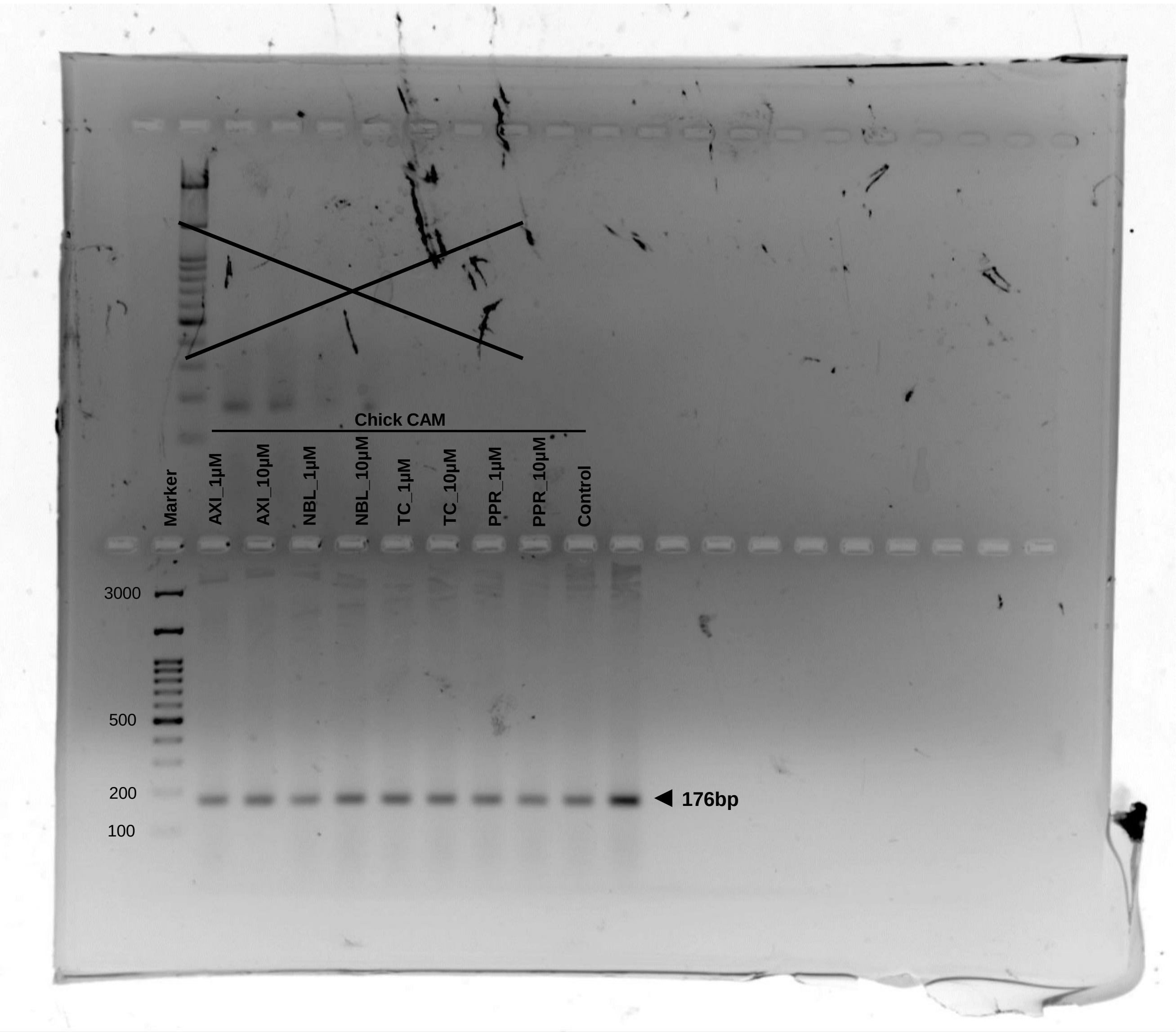

Chick CAM standalone tissue: GAPDH chick primer

Replicate 3

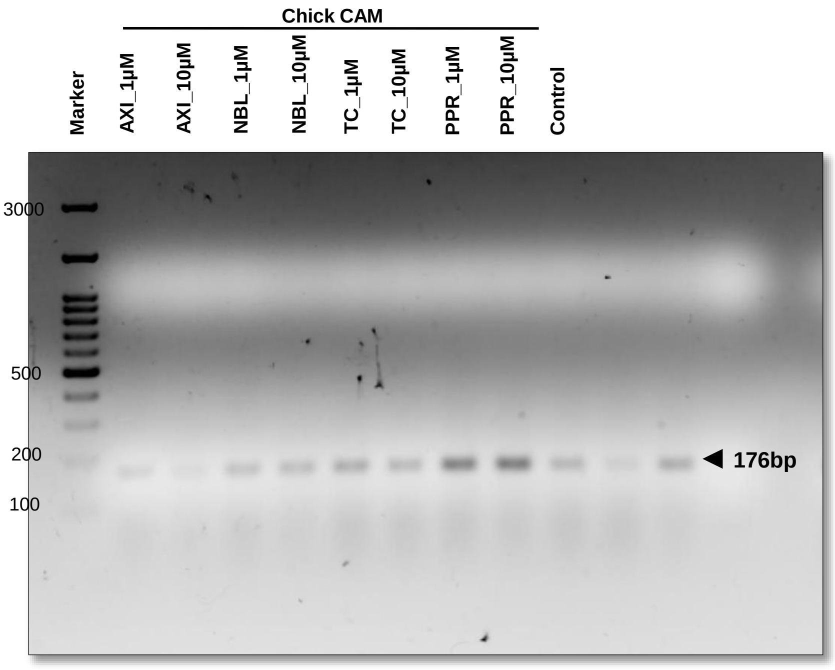

Chick CAM U87 xenograft tissue: VEGF-A chick primer

Figure 10A (Representative Blot), Replicate 1

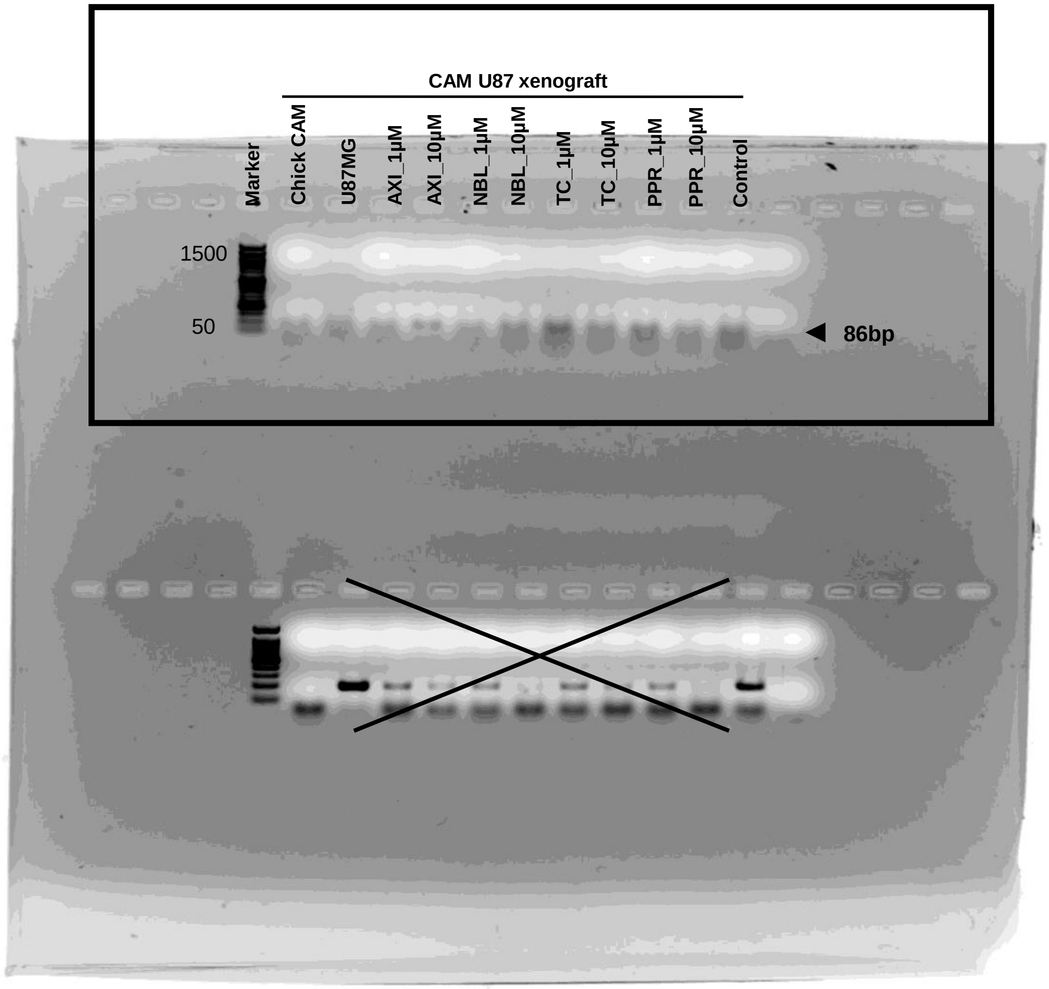

Black box – Representative gel image incorporated in the text

Chick CAM U87 xenograft tissue: VEGF-A chick primer

Replicate 2

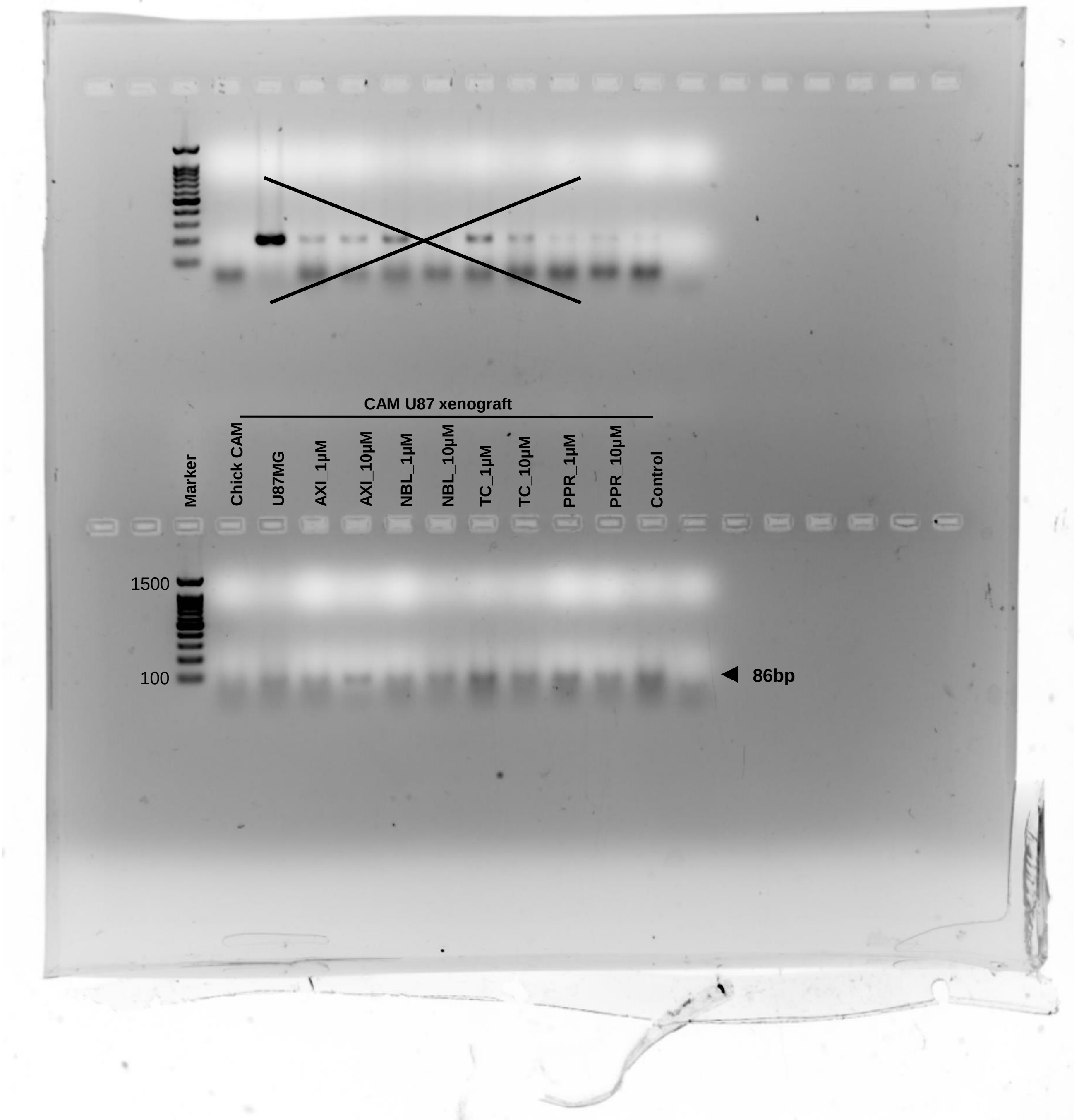

Chick CAM U87 xenograft tissue: VEGF-A chick primer

Replicate 3

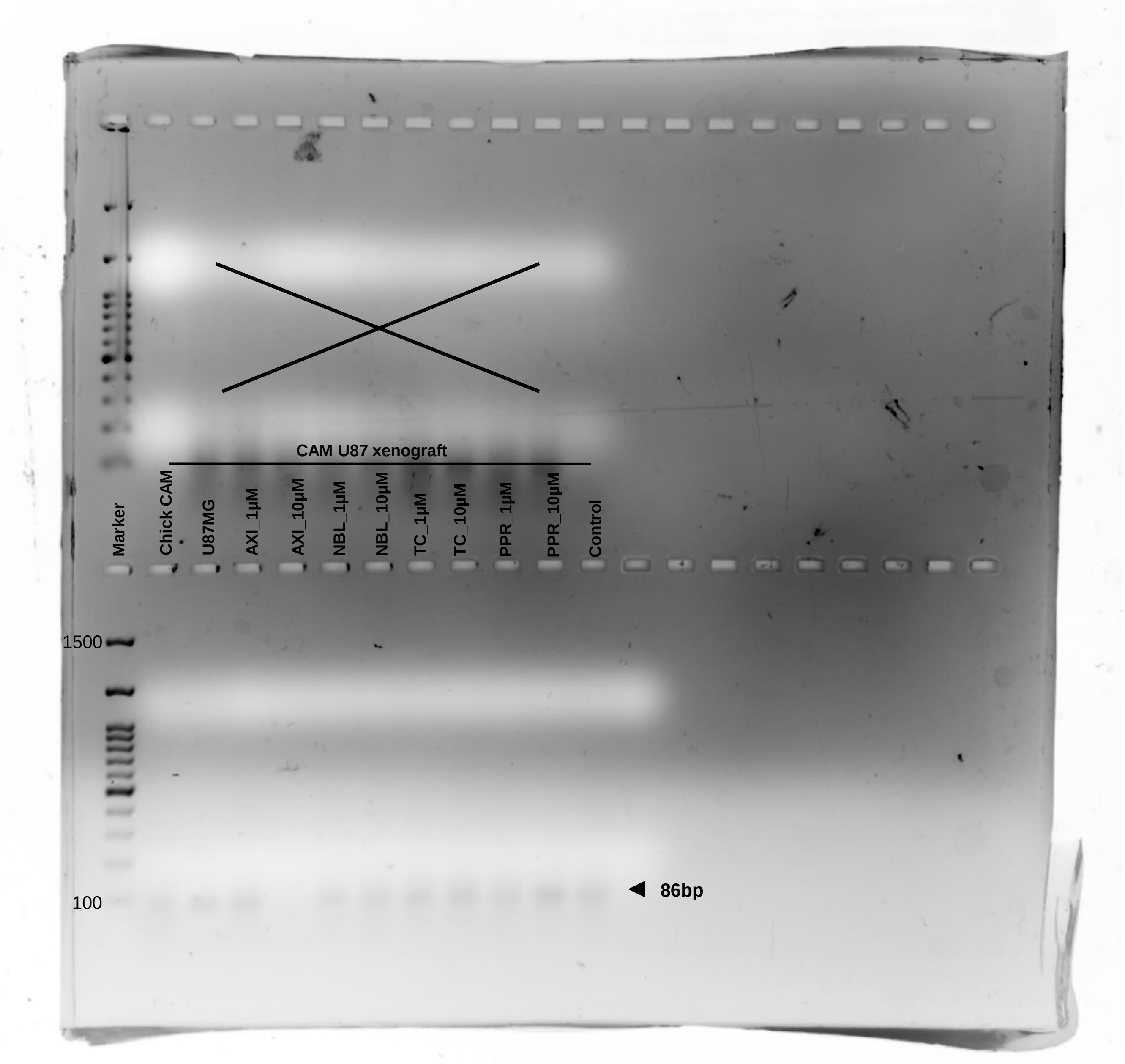

Chick CAM U87 xenograft tissue: VEGFR-2 chick primer

Figure 10A (Representative Blot), Replicate 1

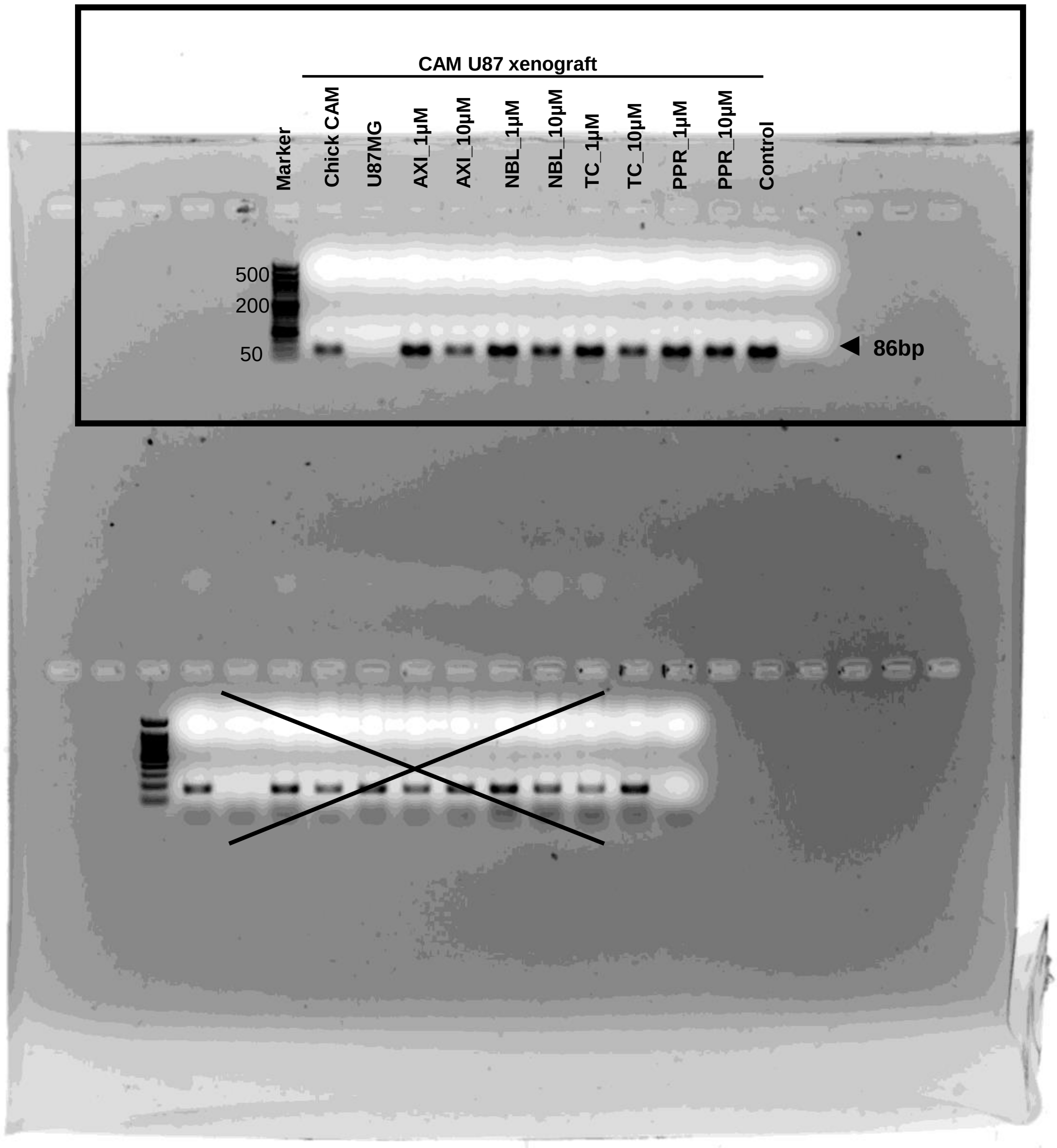

Black box – Representative gel image incorporated in the text

Chick CAM U87 xenograft tissue: VEGFR-2 chick primer

Replicate 2

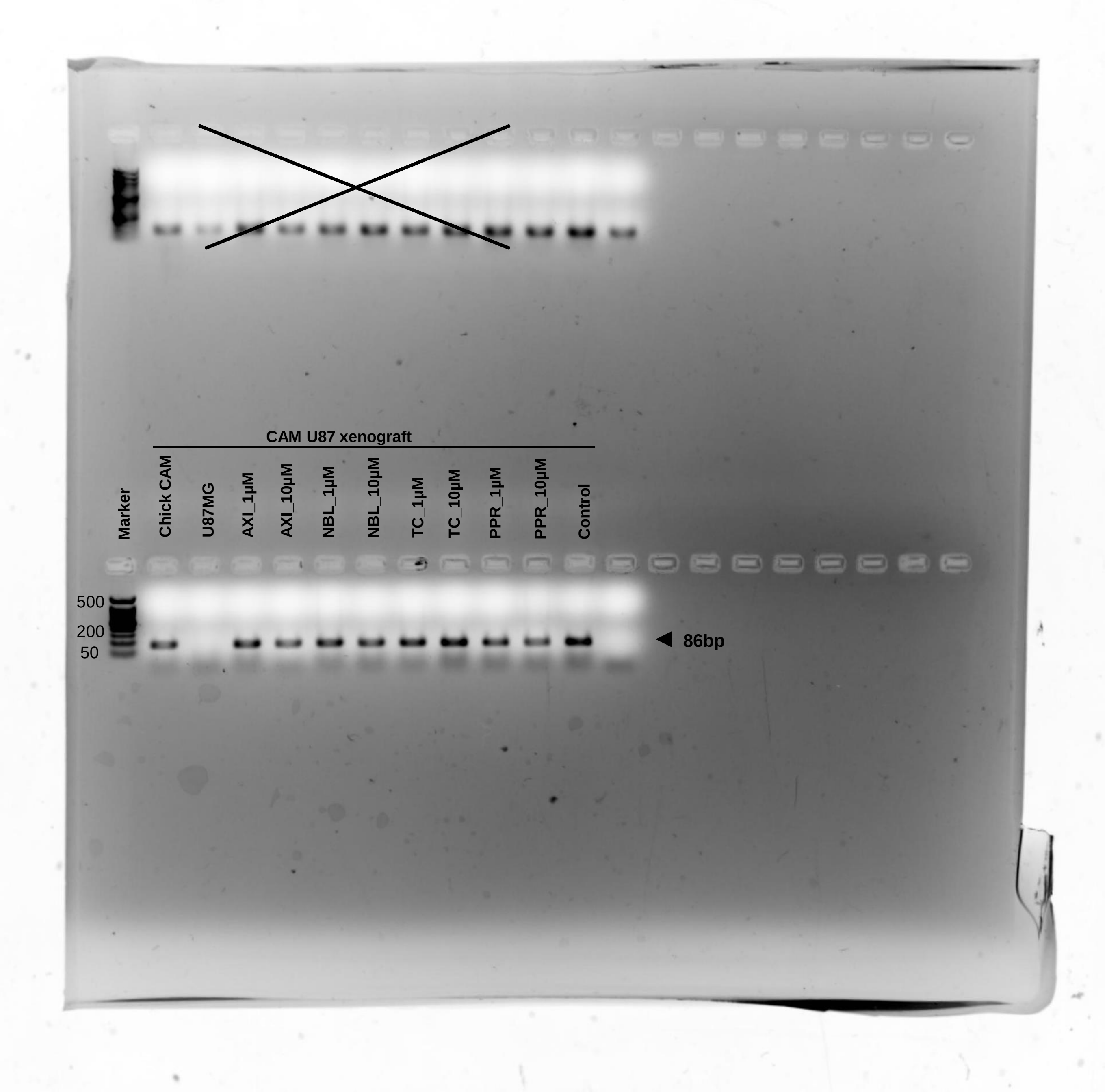

Chick CAM U87 xenograft tissue: VEGFR-2 chick primer

Replicate 3

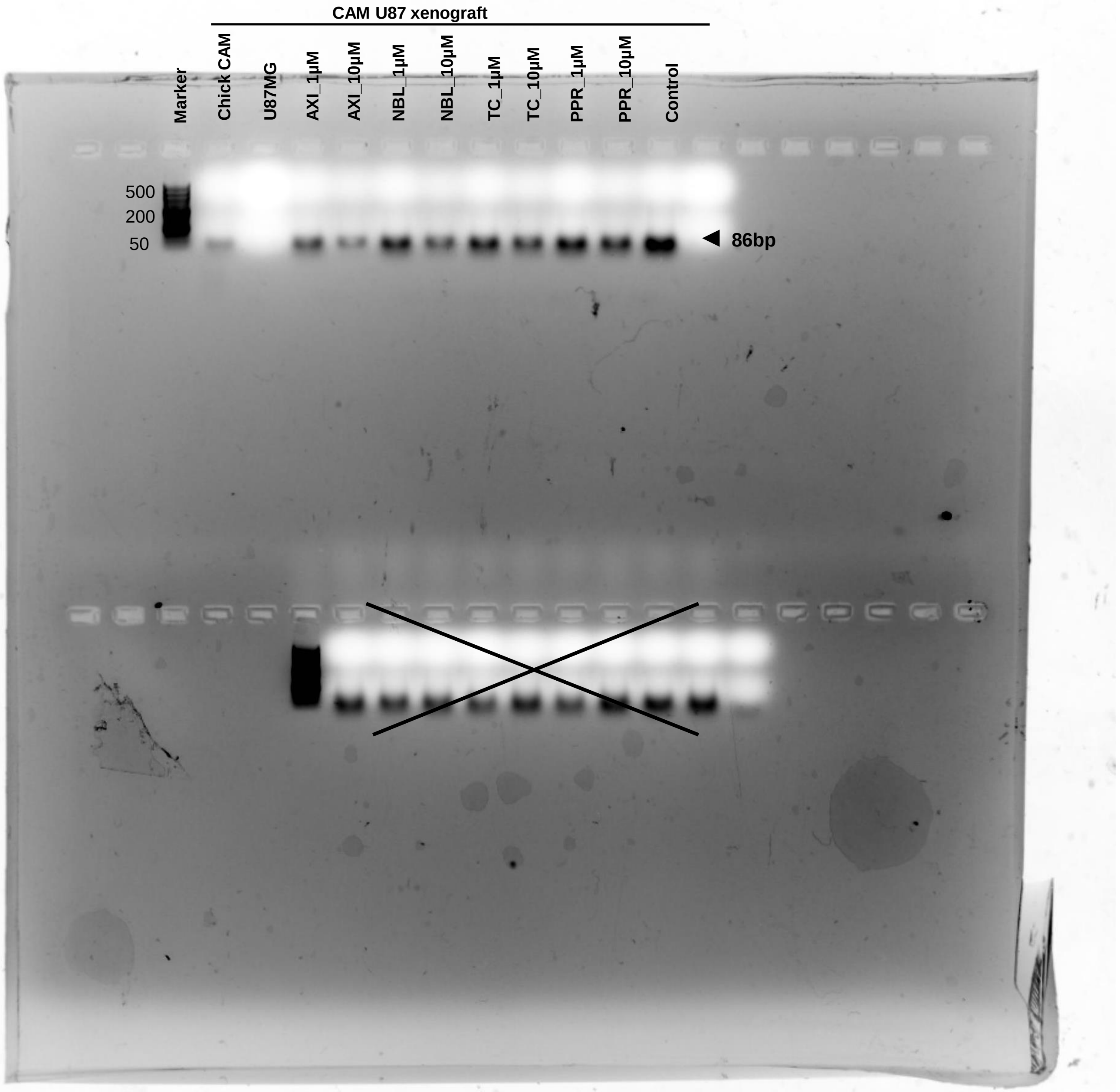

Chick CAM U87 xenograft tissue: GAPDH chick primer

Figure 10A (Representative Blot), Replicate 1

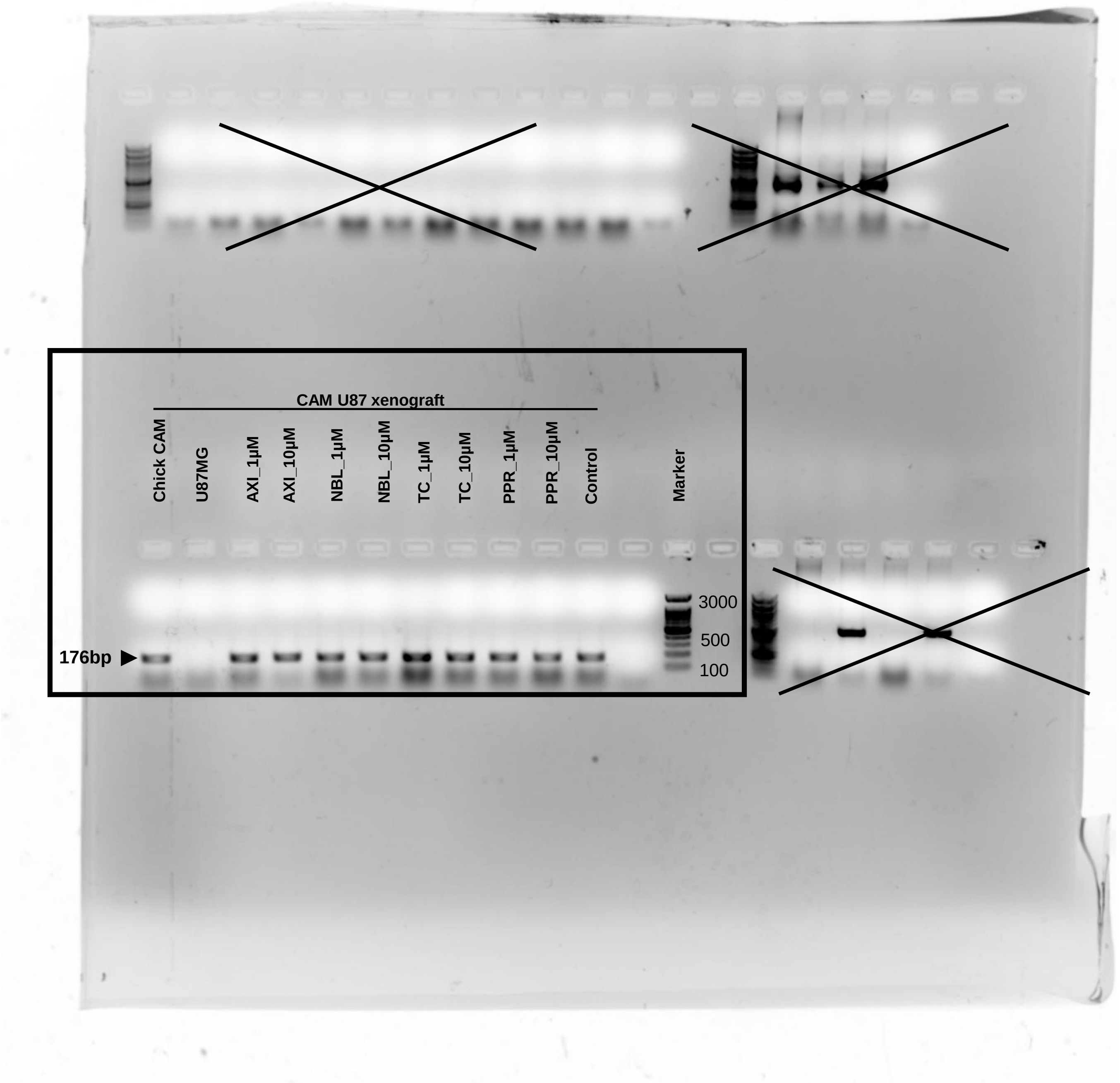

Black box – Representative gel image incorporated in the text

SF6

Chick CAM U87 xenograft tissue: GAPDH chick primer

Replicate 2

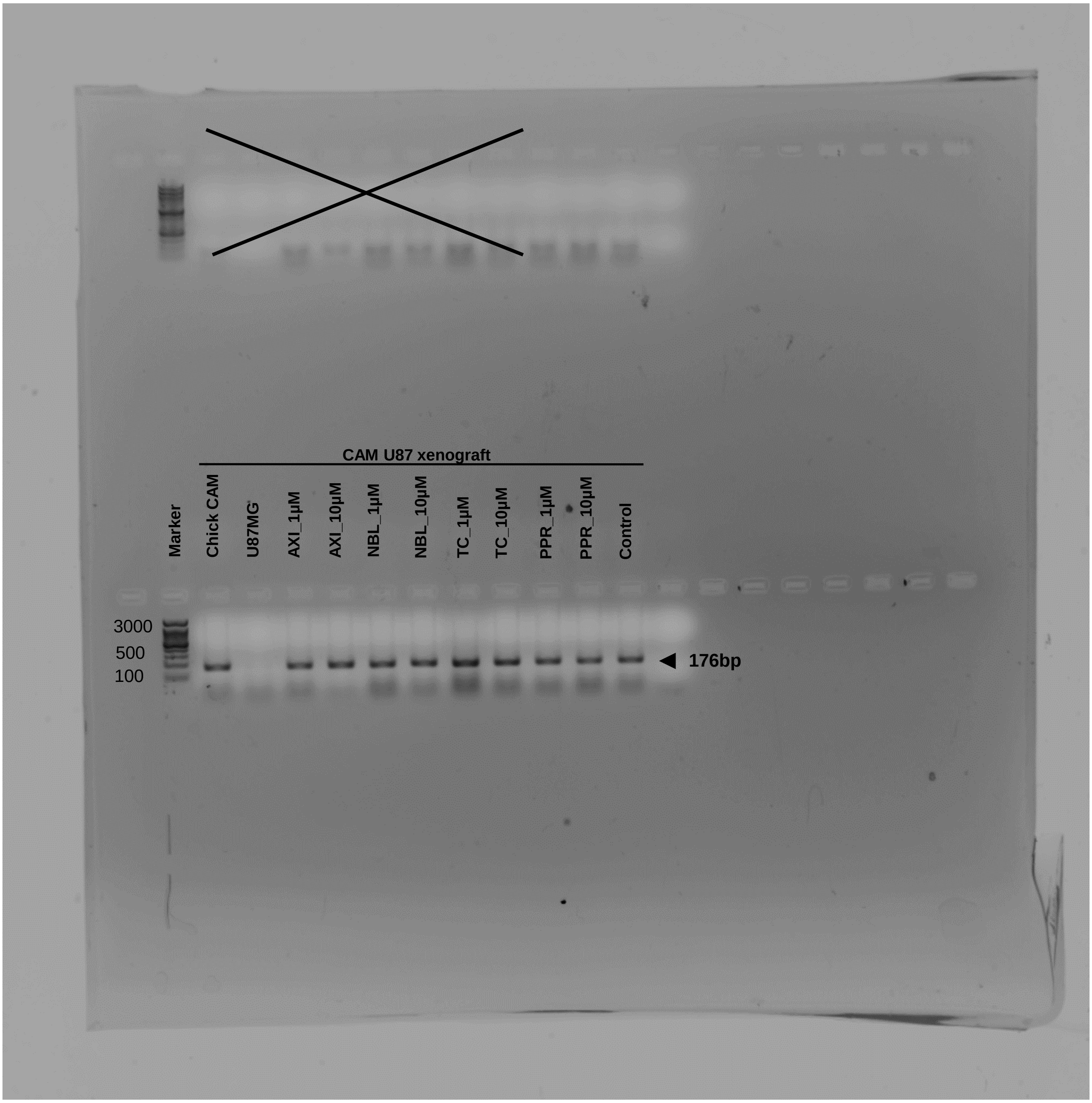

Chick CAM U87 xenograft tissue: GAPDH chick primer

Replicate 3

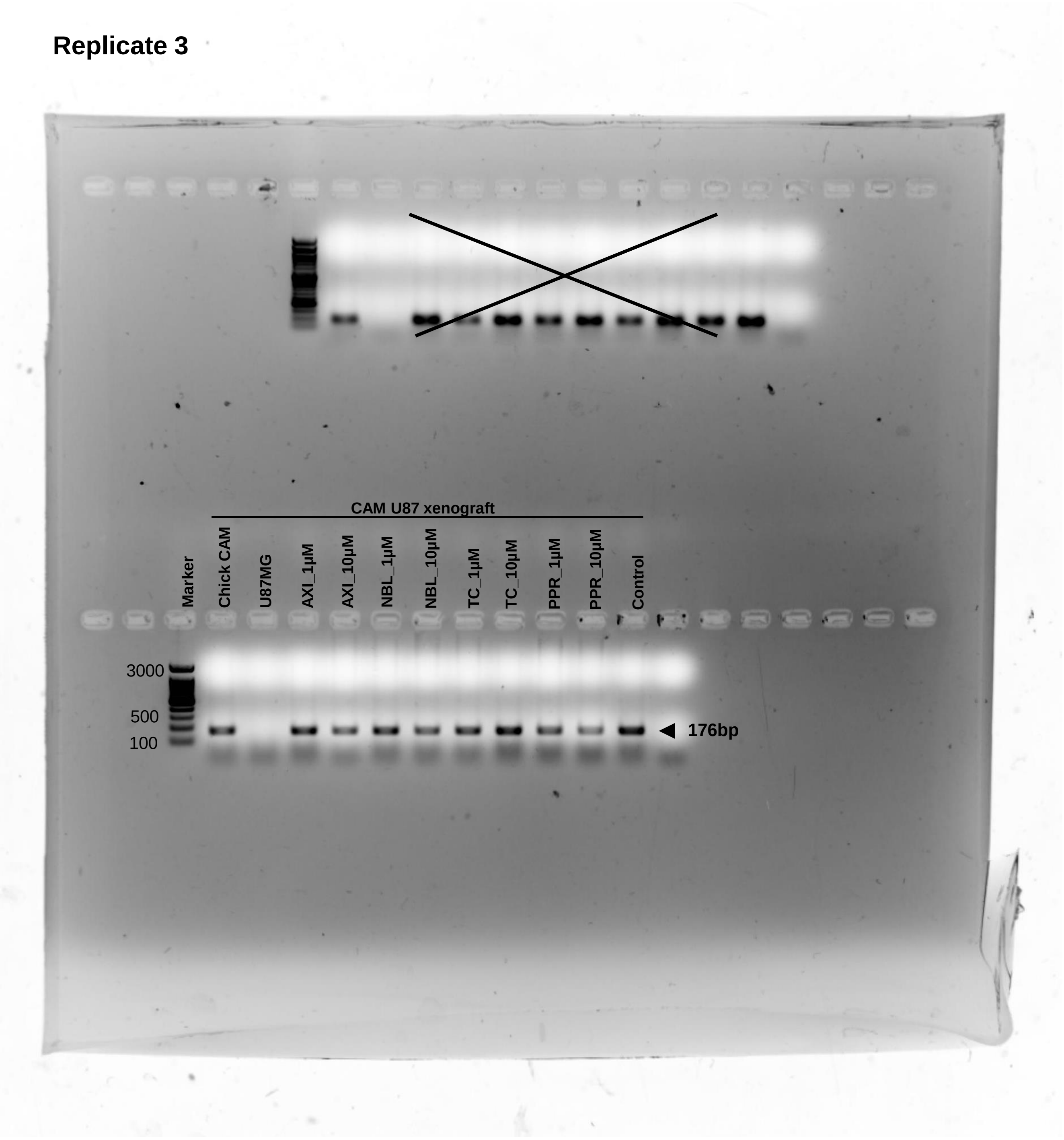

Chick CAM U87 xenograft tissue: VEGF-A Human primer

Figure 10A (Representative Blot), Replicate 1

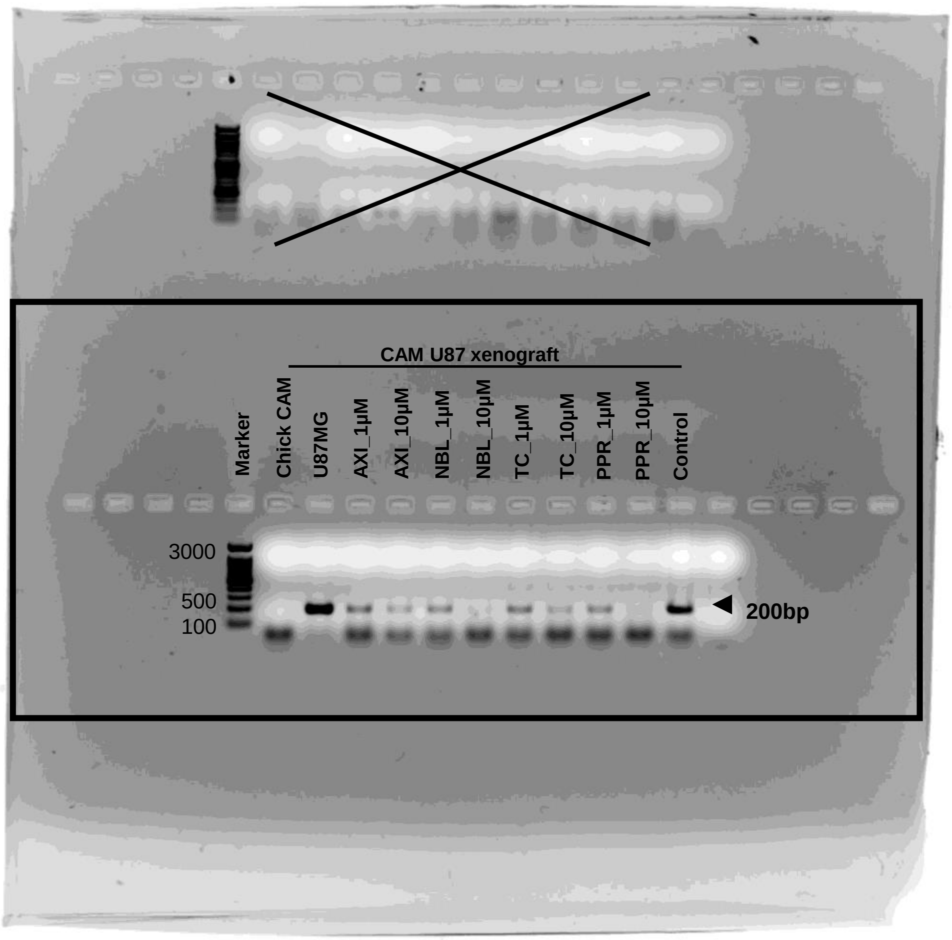

Black box – Representative gel image incorporated in the text

SF7

Chick CAM U87 xenograft tissue: VEGF-A Human primer

Replicate 2

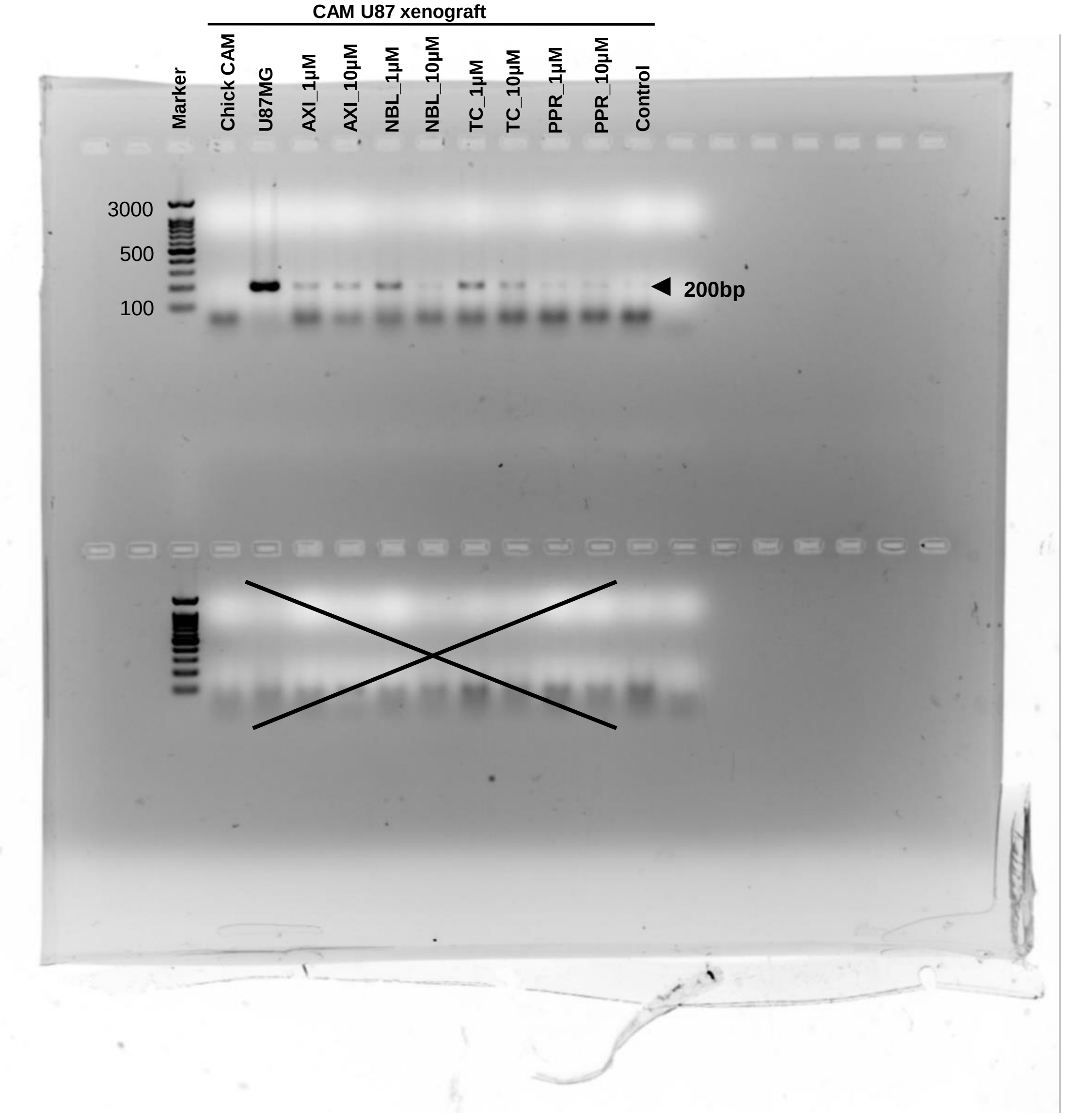

Chick CAM U87 xenograft tissue: VEGFR-2 Human primer

Figure 10A (Representative Blot), Replicate 1

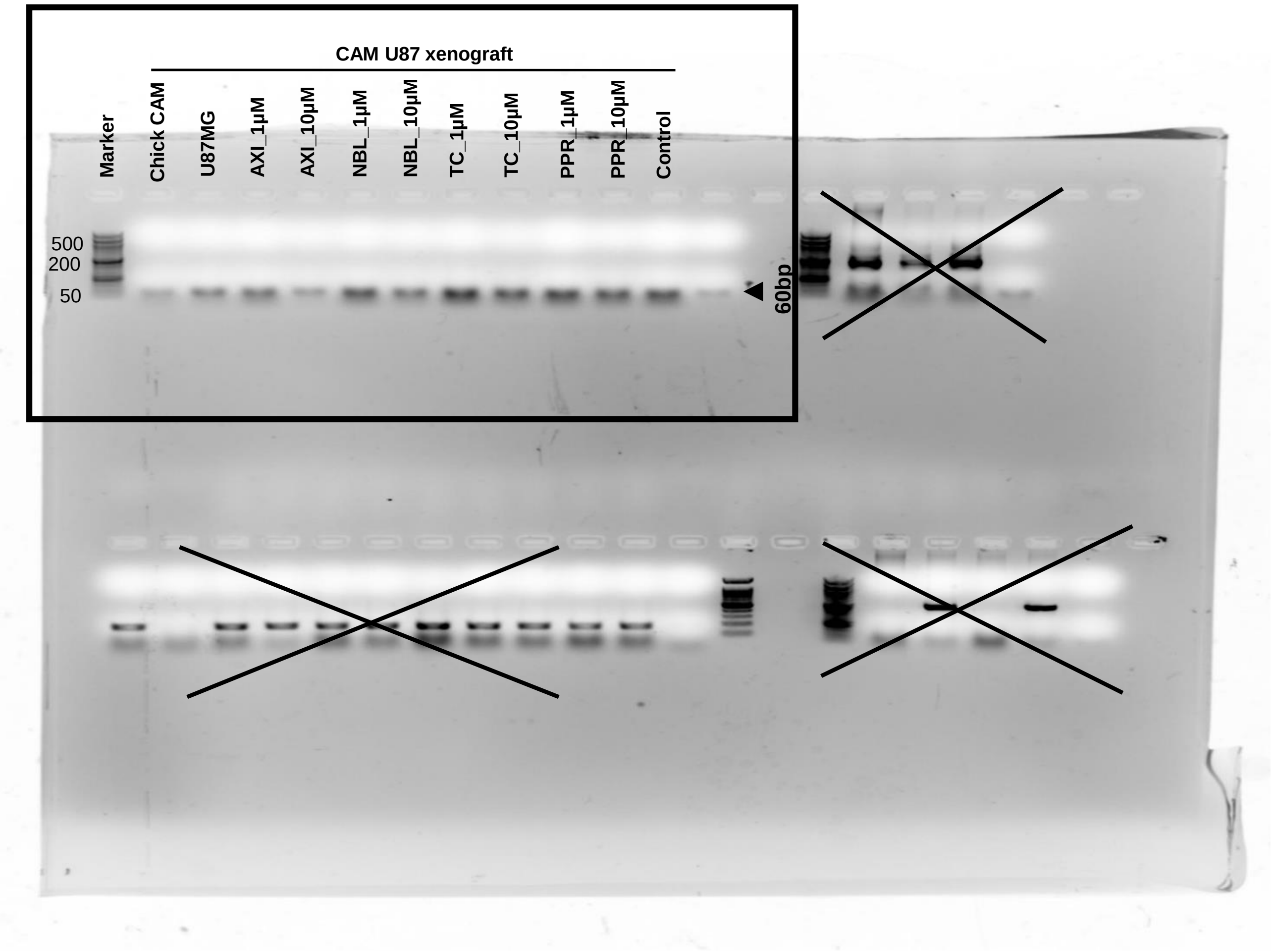

Black box – Representative gel image incorporated in the text

SF8

Chick CAM U87 xenograft tissue: VEGFR-2 Human primer

Replicate 2

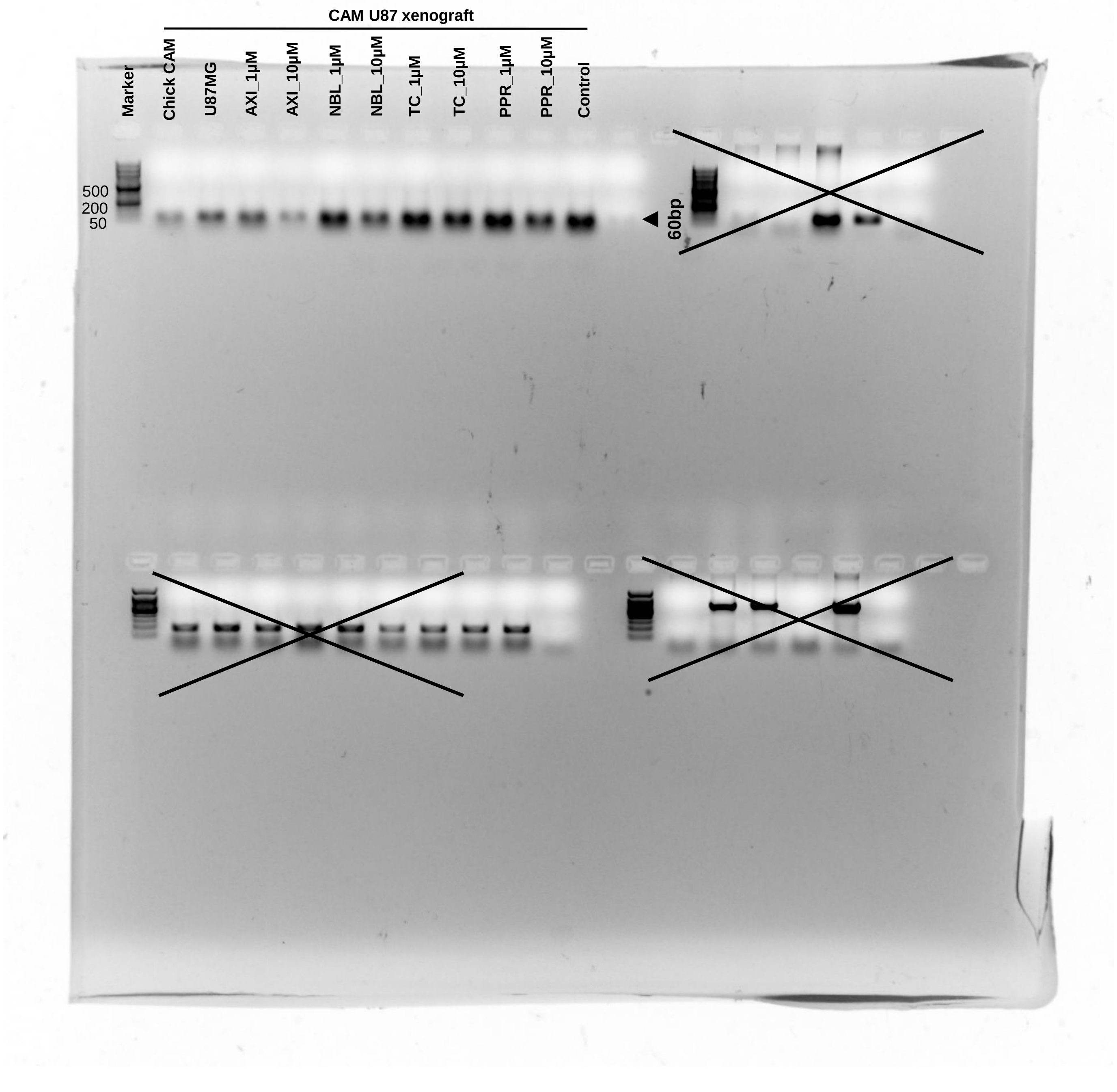

SF8

Chick CAM U87 xenograft tissue: VEGFR-2 Human primer

Replicate 3

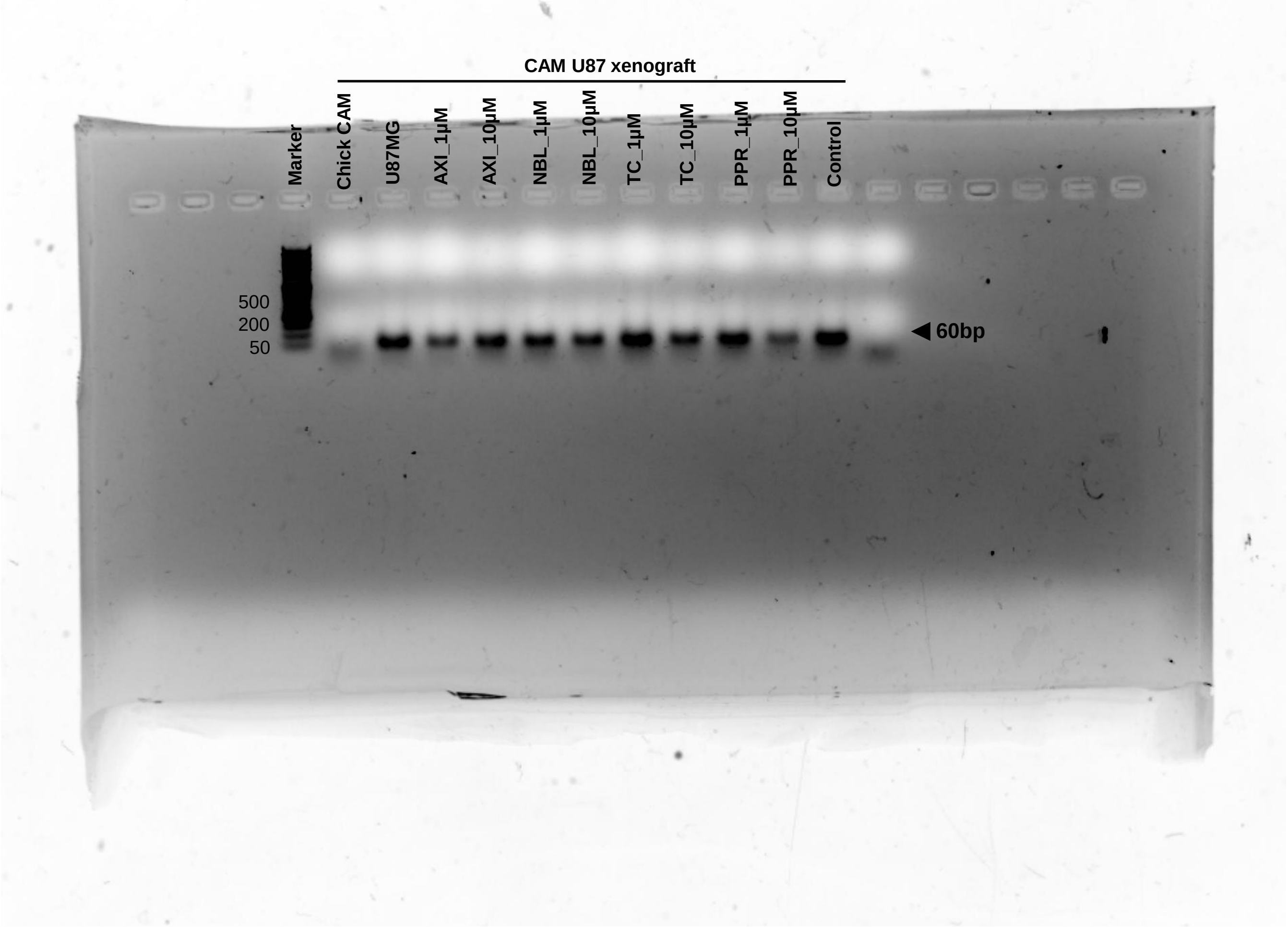

SF8

Chick CAM U87 xenograft tissue: VEGFR-2 Human primer

Replicate 4

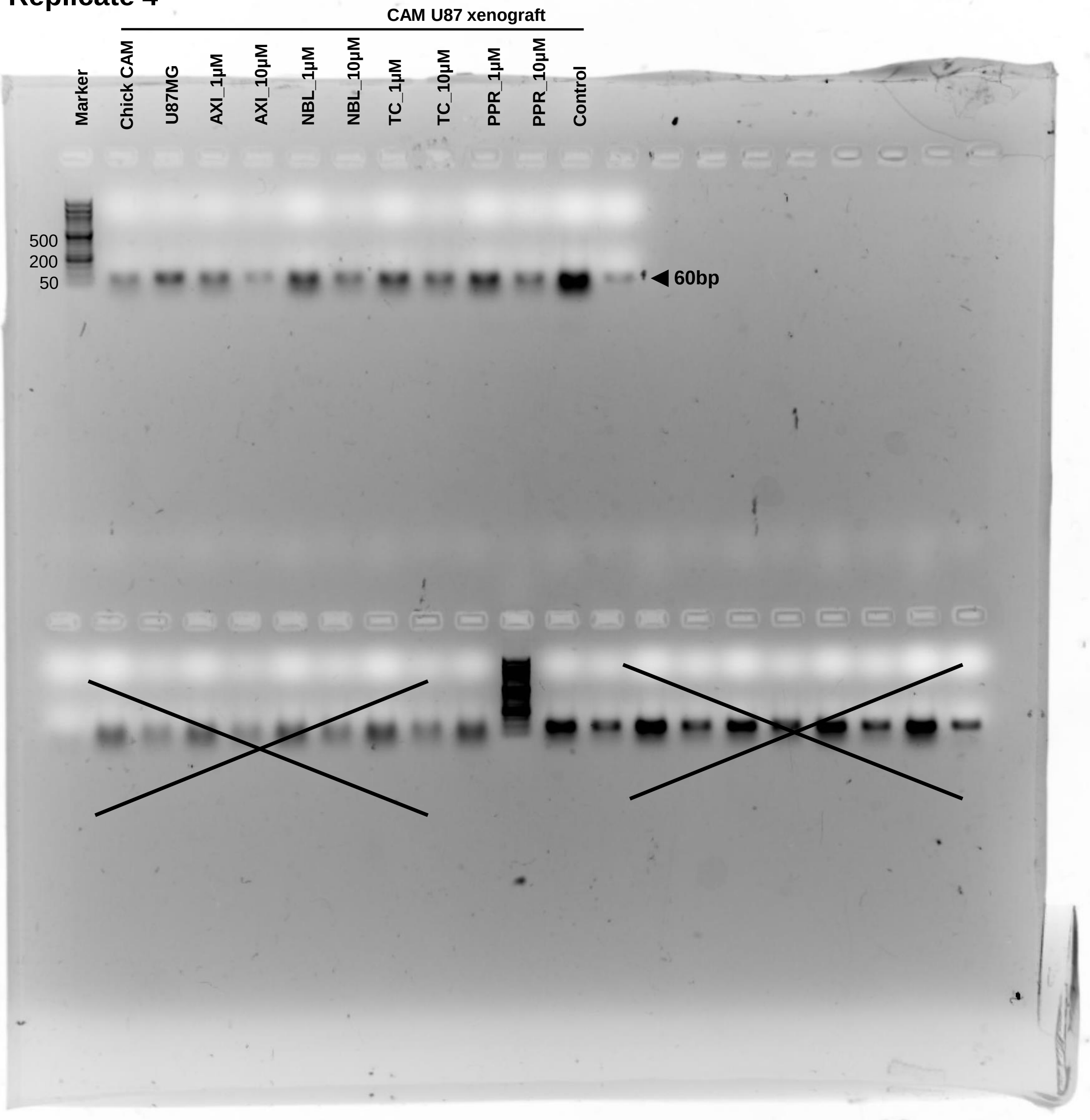

Chick CAM U87 xenograft tissue: GAPDH Human primer

Figure 10A (Representative Blot)

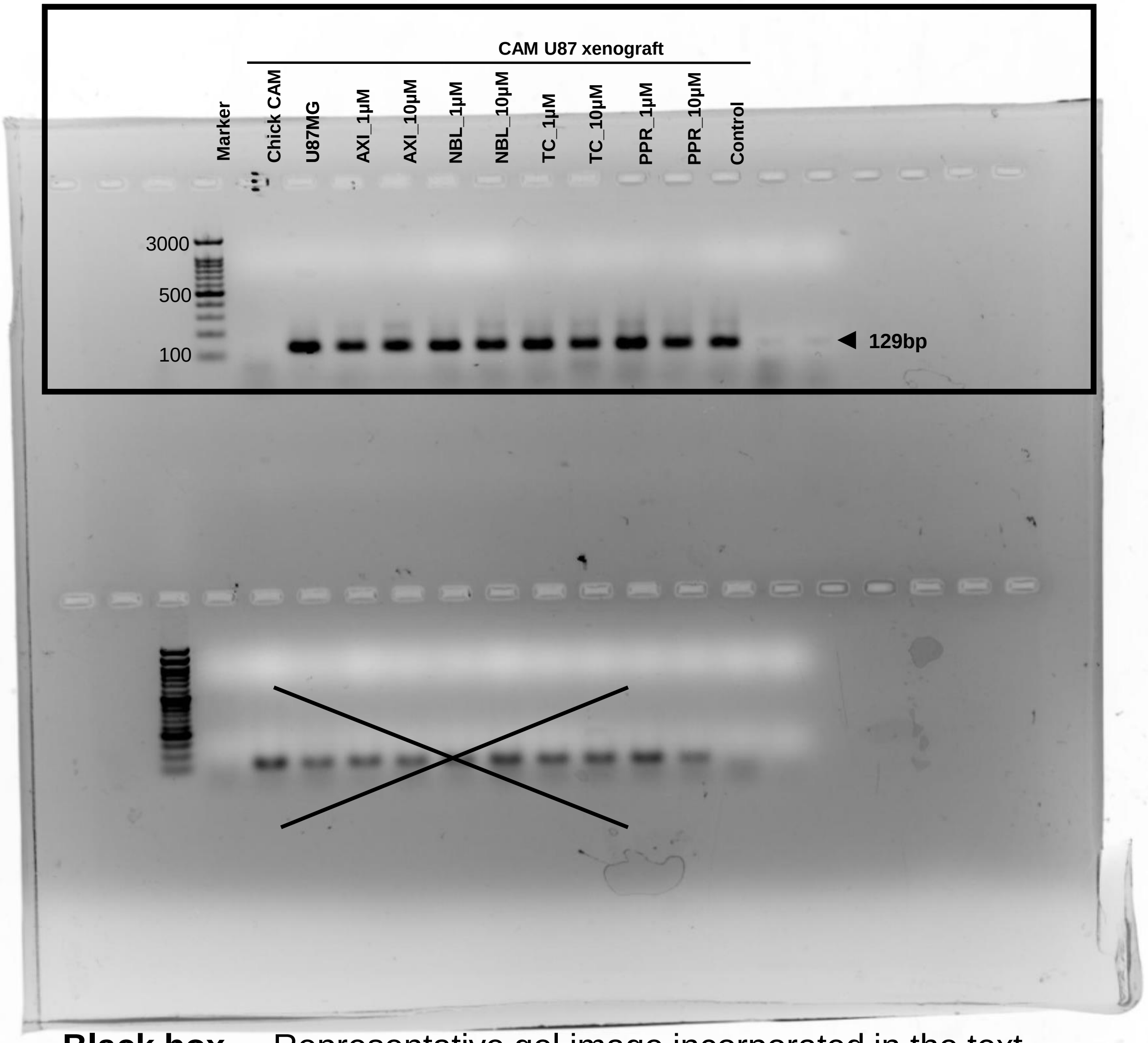

Black box – Representative gel image incorporated in the text
