# Supplementary material for: In vivo antiangiogenic effect of nimbolide, trans-chalcone and piperine for use against glioblastoma
Source: BMC Cancer. 2023 Nov 30;23:1173. doi: 10.1186/s12885-023-11625-4 (PMC10691152; doi:10.1186/s12885-023-11625-4)
Supplement: Supplementary file 1 — Additional file 1. [file 12885_2023_11625_MOESM1_ESM.zip › Supplementary Figs_20230602 (2).pptx]

## Slide 1
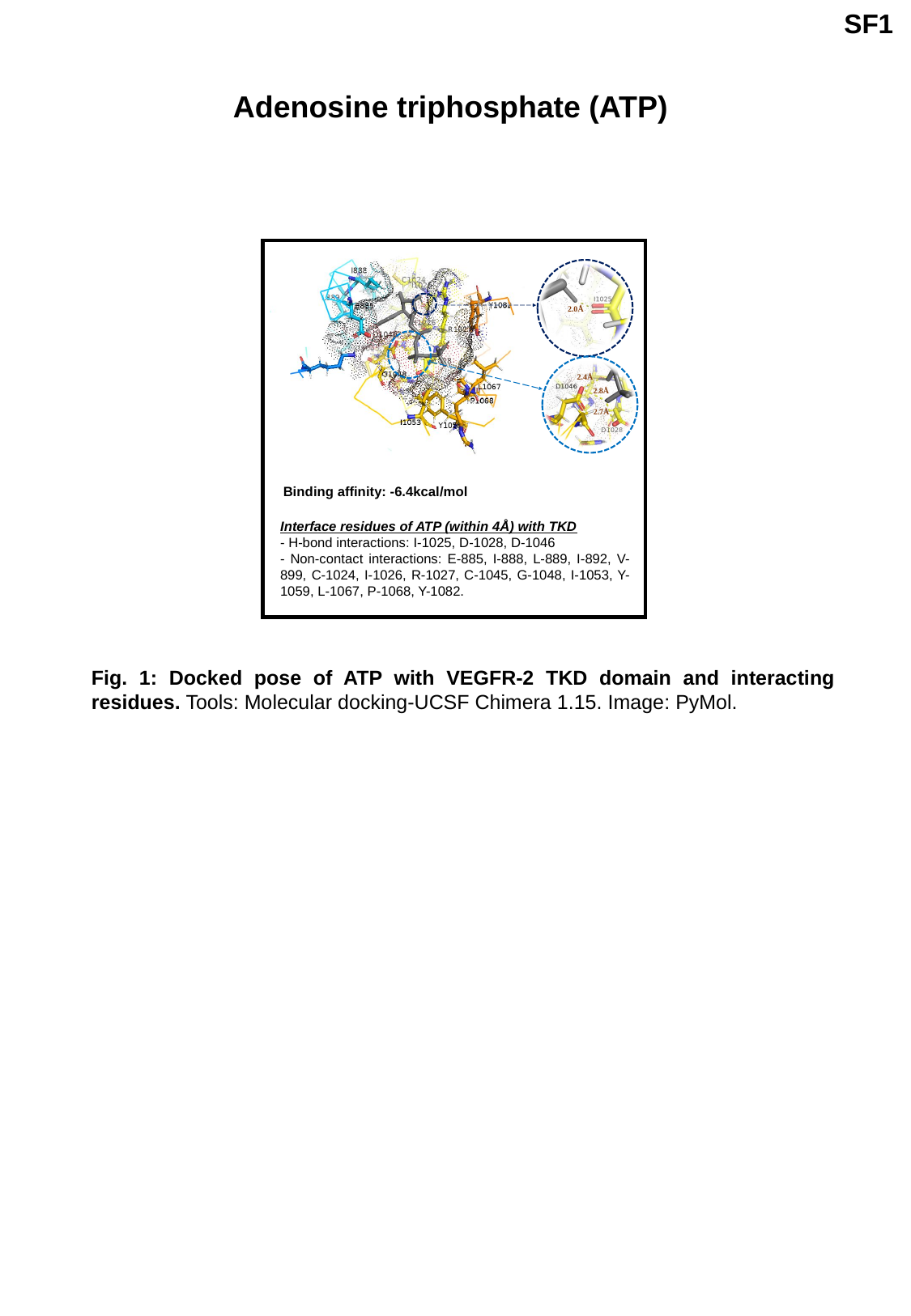

SF1
 Adenosine triphosphate (ATP)
Binding affinity: -6.4kcal/mol
Interface residues of ATP (within 4Å) with TKD
- H-bond interactions: I-1025, D-1028, D-1046
- Non-contact interactions: E-885, I-888, L-889, I-892, V-899, C-1024, I-1026, R-1027, C-1045, G-1048, I-1053, Y-1059, L-1067, P-1068, Y-1082.
Fig. 1: Docked pose of ATP with VEGFR-2 TKD domain and interacting residues. Tools: Molecular docking-UCSF Chimera 1.15. Image: PyMol.

## Slide 2
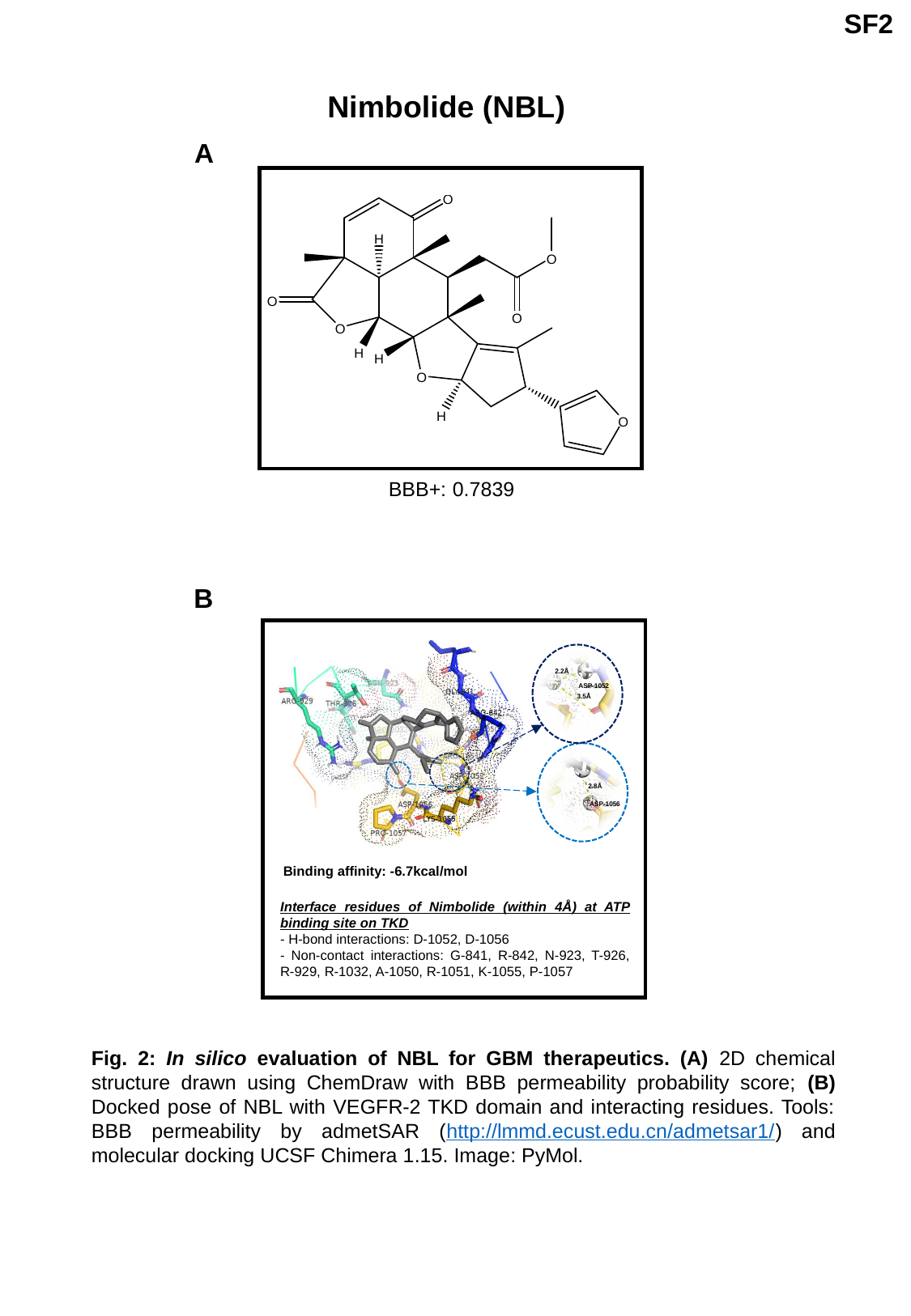

SF2
Nimbolide (NBL)
A
 BBB+: 0.7839
B
2.2Å
ASP-1052
3.5Å
2.8Å
ASP-1056
Binding affinity: -6.7kcal/mol
Interface residues of Nimbolide (within 4Å) at ATP binding site on TKD
- H-bond interactions: D-1052, D-1056
- Non-contact interactions: G-841, R-842, N-923, T-926, R-929, R-1032, A-1050, R-1051, K-1055, P-1057
Fig. 2: In silico evaluation of NBL for GBM therapeutics. (A) 2D chemical structure drawn using ChemDraw with BBB permeability probability score; (B) Docked pose of NBL with VEGFR-2 TKD domain and interacting residues. Tools: BBB permeability by admetSAR (http://lmmd.ecust.edu.cn/admetsar1/) and molecular docking UCSF Chimera 1.15. Image: PyMol.

## Slide 3
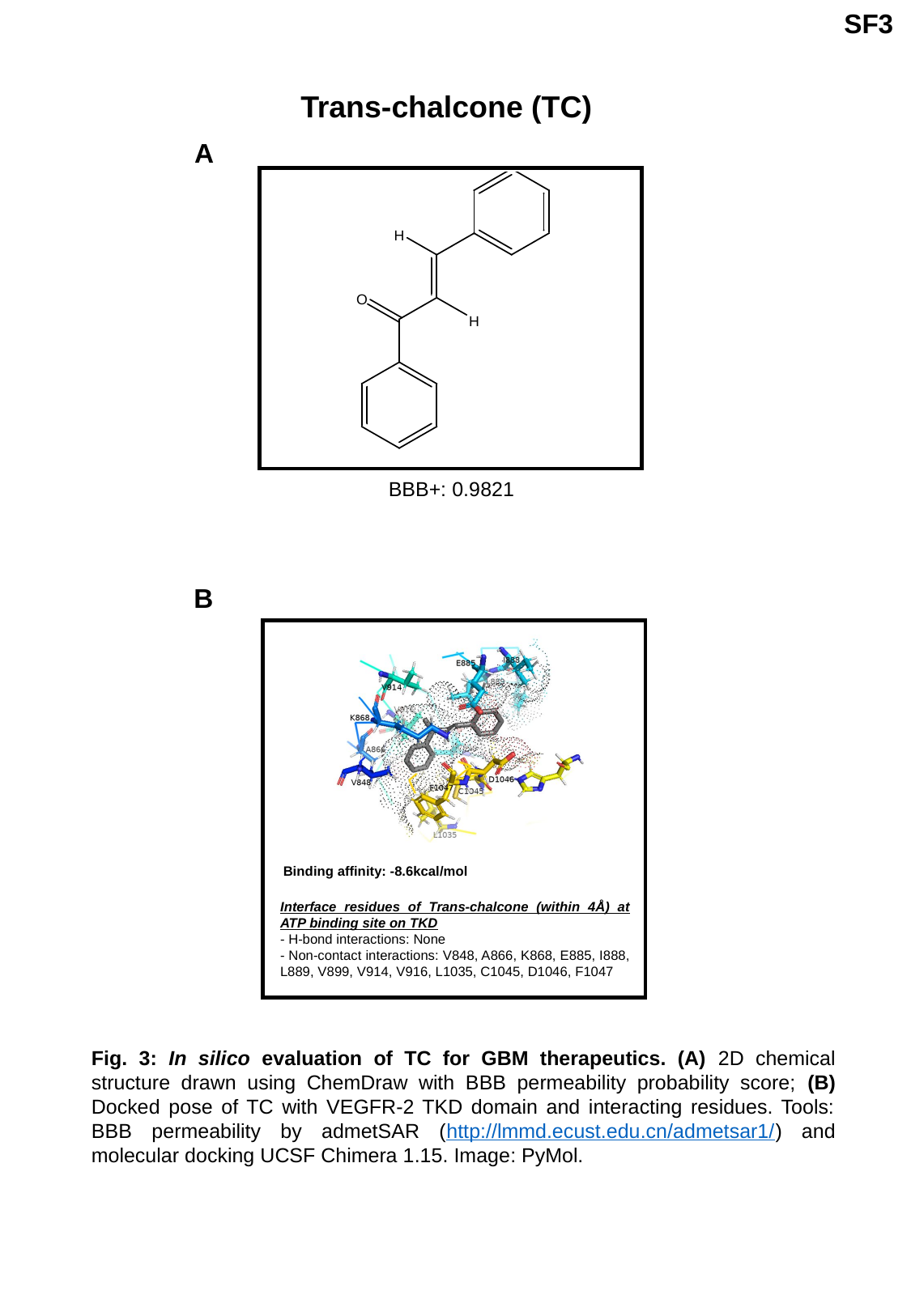

SF3
Trans-chalcone (TC)
A
 BBB+: 0.9821
B
Binding affinity: -8.6kcal/mol
Interface residues of Trans-chalcone (within 4Å) at ATP binding site on TKD
- H-bond interactions: None
- Non-contact interactions: V848, A866, K868, E885, I888, L889, V899, V914, V916, L1035, C1045, D1046, F1047
Fig. 3: In silico evaluation of TC for GBM therapeutics. (A) 2D chemical structure drawn using ChemDraw with BBB permeability probability score; (B) Docked pose of TC with VEGFR-2 TKD domain and interacting residues. Tools: BBB permeability by admetSAR (http://lmmd.ecust.edu.cn/admetsar1/) and molecular docking UCSF Chimera 1.15. Image: PyMol.

## Slide 4
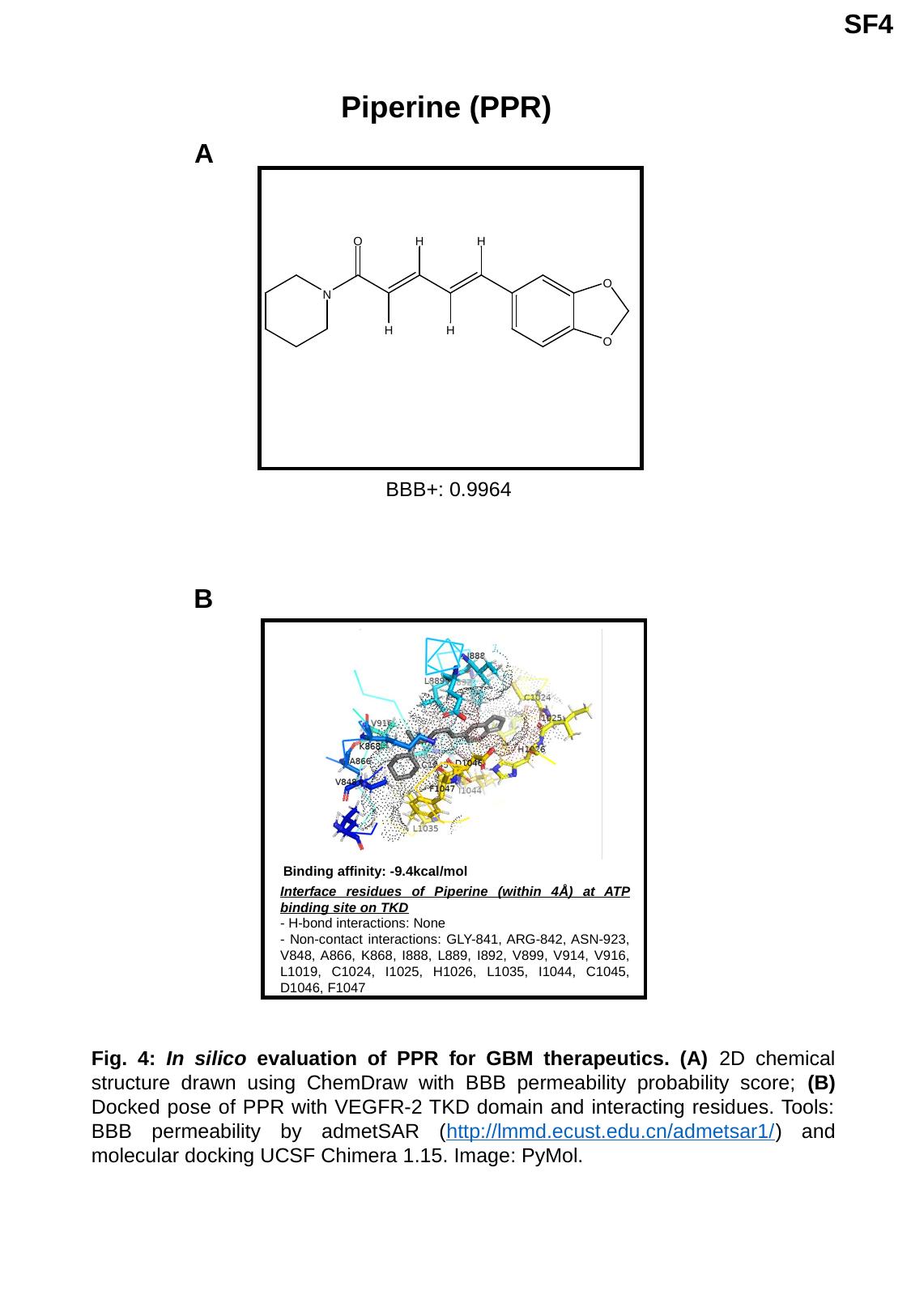

SF4
Piperine (PPR)
A
BBB+: 0.9964
B
Binding affinity: -9.4kcal/mol
Interface residues of Piperine (within 4Å) at ATP binding site on TKD
- H-bond interactions: None
- Non-contact interactions: GLY-841, ARG-842, ASN-923, V848, A866, K868, I888, L889, I892, V899, V914, V916, L1019, C1024, I1025, H1026, L1035, I1044, C1045, D1046, F1047
Fig. 4: In silico evaluation of PPR for GBM therapeutics. (A) 2D chemical structure drawn using ChemDraw with BBB permeability probability score; (B) Docked pose of PPR with VEGFR-2 TKD domain and interacting residues. Tools: BBB permeability by admetSAR (http://lmmd.ecust.edu.cn/admetsar1/) and molecular docking UCSF Chimera 1.15. Image: PyMol.
