# Supplementary material for: In vivo antiangiogenic effect of nimbolide, trans-chalcone and piperine for use against glioblastoma
Source: BMC Cancer. 2023 Nov 30;23:1173. doi: 10.1186/s12885-023-11625-4 (PMC10691152; doi:10.1186/s12885-023-11625-4)
Supplement: Supplementary file 1 — Additional file 1. [file 12885_2023_11625_MOESM1_ESM.zip › Supplementary Table ST1_20230601.docx]

**Supplementary Table ST1: List of primers used in the study along with their sequence and annealing temperatures**

| **Primer** | **Sequence (5’-3’)** | **Amplicon size** | **Annealing temp.** | **Source** | **Reference** |
| --- | --- | --- | --- | --- | --- |
| VEGF-A | FP: CAA TTG AGA CCC TGG TGG AC  RP: TCT CAT CAG AGG CAC ACA GG | 86 bp | 60 | *Gallus gallus* | **(Gheorghescu *et al.*, 2015)** |
| VEGFR-2 | FP: GCC AAC TCT ATG GCA GAA GC  RP: CTG AAC ACC ATG CCA CTG TC | 86 bp | 60 | *Gallus gallus* | **(Gheorghescu *et al.*, 2015)** |
| GAPDH | FP: CCT CTC TGG CAA AGT CCA AG  RP: GGT CAC GCT CCT GGA AGA TA | 176 bp | 62 | *Gallus gallus* | **(Gheorghescu *et al.*, 2015)** |
| VEGF-A | FP: CTTGCCTTGCTGCTCTACC  RP: CACACAGGATGGCTTGAAG | 200 bp | 56 | *Human* | **(Niki *et al.*, 2000)** |
| VEGFR-2 | FP: ATTCCTCCCCCGCATCA  RP: GCTCGTTGGCGCACTCTT | 60 bp | 58 | *Human* | **(Domingues *et al.*, 2011)** |
| GAPDH | FP: GTCTCCTCTGACTTCAACAGCG  RP: ACCACCCTGTTGCTGTAGCCAA | 129 bp | 62 | *Human* | **(Nabokina *et al.*, 2017)** |
